# Imaging brain inflammation and blood brain barrier permeability in neurological and psychiatric diseases: a review

**DOI:** 10.1186/s12974-025-03598-x

**Published:** 2025-11-19

**Authors:** Zanetta Kovbasyuk, Eden Tefera, Chenyang Li, Steven H. Baete, Claude Steriade

**Affiliations:** 1 Department of Neurology, New York University Langone Medical Center, 223 East 34Th Street, New York, NY 10016 USA; 2 Center for Advanced Imaging Innovation and Research (CAI2R), Department of Radiology, New York University Langone Medical Center, New York, NY 10016 USA; 3 Vilcek Institute of Graduate Biomedical Sciences, New York University School of Medicine, New York, NY 10016 USA; 4 Neuroscience Institute, New York University School of Medicine, New York, NY 10016 USA

## Abstract

Neuroinflammation involving glial cell activation and BBB dysfunction has increasingly been recognized as a key feature of neuropsychiatric disorders. In vivo imaging methods, particularly translocator protein positron emission tomography (TSPO-PET) and dynamic contrast-enhanced magnetic resonance imaging (DCE-MRI), have advanced our understanding of glial activation and BBB permeability in conditions such as Alzheimer’s disease, Parkinson’s disease, epilepsy, multiple sclerosis, Huntington’s disease, schizophrenia, and depression. We present key findings from the clinical application of these imaging modalities and highlight critical methodological challenges—including variability in study protocols, tracer selection, input function derivation, and parameter estimation—that currently limit cross-study comparability and clinical translation. TSPO-PET and DCE-MRI provide valuable clinical insights on the inflammatory mechanisms contributing to CNS disease at various disease stages. Future methodological standardization, co-localization studies, and longitudinal multi-modal applications will be crucial for using these tools as markers of disease in the context of immune interventions in at-risk populations.

**Supplementary Information:**

The online version contains supplementary material available at 10.1186/s12974-025-03598-x.

## Introduction

Neuroinflammation, involving blood brain barrier (BBB) dysfunction and activation of microglia and reactive astrocytes has long been implicated in brain disorders [1, 2]. Over the past 25 years, advanced imaging methods have investigated neuroinflammatory process for diagnostic and therapeutic monitoring of many neuropsychiatric conditions [3, 4]. This Review focuses on neuroimaging applications to measure brain inflammation in vivo in central nervous system (CNS) disorders including Alzheimer’s disease (AD), Parkinson’s Disease (PD), epilepsy, multiple sclerosis (MS), Huntington’s disease (HD), schizophrenia (Scz), and depression. Specifically, we examine neuroimaging methods targeting (1) CNS immune cell activation using translocator protein (TSPO) positron emission tomography (PET) imaging and (2) BBB dysfunction using dynamic contrast-enhanced magnetic resonance imaging (DCE-MRI).

### TSPO-PET imaging

Microglia are central to the brain’s immune system and are typically activated early following neuronal insults, [1] initiating astrocyte activation through proinflammatory mediators which typically serves a neurosupportive role [5]. Microglial dysfunction can also be triggered by environmental risk factors and inherent susceptibility [6, 7]. However, sustained homeostatic glial activation can become pathogenic. The 18-kDA TSPO is expressed on the outer mitochondrial membranes of activated microglia, and recently has been observed in reactive astrocytes, endothelium and macrophages in the context of pathology [8]. Therefore, TSPO expression is a biomarker of inflammatory load reflecting microglial activity, reactive astrogliosis and broader neuroinflammation. TSPO-PET has been used to characterize neuroinflammatory processes across several CNS disorders in vivo, [9] but emerging evidence challenges whether the TSPO signal reflects changes in reactivity or cell density [10] (Table 1). We therefore refer to TSPO-PET outcomes as “glial activation” in this review.

**Table 1:** TSPO-PET review of quantification methods


PET imaging measures the distribution of ligands labeled with positron-emitting isotopes (such as ^18^F, ^11^C, or ^15^O) in vivo via detection of annihilation photons. To quantify TSPO binding, dynamic scans are typically paired with continuous arterial blood sampling to measure radiotracer concentration in the blood over time. It also provides a measure of tracer metabolism, permitting quantification of the parent tracer (intact tracer) and its metabolites [9]
Kinetic models of tracer uptake and binding are derived by integrating the blood and PET data. The blood data serves as the input function for kinetic models and describes how the tracer enters the tissue. PET-derived time-activity curves describe tracer accumulation and clearance in tissue. The PET signal can also be adjusted for the metabolized components of the blood, resulting in a signal derived from tracer binding that is still in its active, intact form. One common model, the two-tissue compartment model (2TCM), estimates binding parameters including non-displaceable binding potential (BP_ND_), total distribution volume (V_T_) and transport rate constants (K1- k4) [11]. VT reflects both specifically bound (V_S_) and free plus non-specifically bound radioligand (V_ND_) [11]. BPND captures target density and ligand affinity [11]. To account for high vascular TSPO binding, [9] a 2TCM with an irreversible vascular compartment (2TCM-1K) was proposed and found to yield stable parameter estimates for several TSPO ligands [12]. Although arterial sampling is considered gold-standard for TSPO-PET studies, absolute quantification using arterial sampling is challenging due to the technical difficulty of placing an arterial line which may contribute to reported variability in parameter estimates. In addition, TSPO has ubiquitous brain expression, precluding reference-region modeling as is standard for other PET studies. To address this, some studies apply a pseudo-normative region for quantification wherein TSPO density is not altered under pathological conditions [11, 13]. Supervised clustering (SVCA) methods to identify voxels with low specific binding as the reference tissue have also been applied to [^11^C]PK11195 and second-generation ligands [11]. Alternatively, image-derived input function (IDIF) [14] and simultaneous estimation (SIME) have been used as less invasive options to arterial sampling [15].
An example of a TSPO-PET acquisition and processing pipeline is depicted below. A.) PET acquisition with or without arterial blood sampling. B.) Input function derivation from arterial blood with metabolite correction or C.) an alternative input function method. D.) Parameter estimation using a pharmacokinetic model with image and input function data as input. Panel C is reproduced from Schubert et al., Eur J Nucl Med Mol Imaging (2021), licensed under CC BY 4.0

Overview of common TSPO-PET acquisition and quantification methods

Several PET tracers have been developed to image inflammatory load with varying signal specificity (Table 2 and Supplementary Table S1, Additional File 1). The first-generation TSPO ligand, [^11^C]PK11195, showed high levels of non-specific binding and poor signal-to-noise ratio (SNR) [9]. Second-generation tracers have improved sensitivity and specificity, but are affected by a polymorphism (Ala147Thr substitution) of the TSPO gene (rs6971) resulting in three TSPO binding affinity patterns [9]. Subjects without the Ala147Thr substitution are considered high-affinity binders (HAB), whereas heterozygotes are mixed-affinity binders (MAB), and homozygotes are low-affinity binders. Since TSPO-PET uptake cannot reliably distinguish between HAB and MAB, genotyping is essential for interpretation [16]. Third-generation PET tracers aim to reduce rs6971 sensitivity, though in vivo studies are limited [17].

**Table 2:** TSPO radiotracers used in vivo

Tracer name		Challenges
**First Generation Tracers**		
**Short Name**	**Long Name**	
[^11^C]PK11195	1-(2-chlorophenyl)-N-[^11^C]methyl-N-(1-methylpropyl)−3-isoquinoline carboxamide	Low SNR due to [3]: - low brain penetrance - high non-specific binding Challenges in quantification [9]: - High affinity for binding sites in the blood and peripheral plasma proteins - Poor free fraction in plasma
Second Generation Tracers		
[^11^C]PBR28	N-((2-(methoxy-^11^C)-phenyl)methyl)-N-(4-phenoxy-3-pyridinyl)acetamide	As compared to [^11^C]PK11195 [17]:
		- Improved SNR - lower nonspecific binding - higher non-displaceable binding potential (BP_ND_) Challenges in quantification [9]: - Confounded by genetic polymorphism (differential expression of TSPO binding sites between subjects, studies must account for differences in binding affinity by comparing within binding affinity groups or including TSPO status in statistical models [16]) - High affinity for binding at the BBB obscures signal in the brain tissue, hampering identification of a suitable reference tissue - Difficult to obtain accurate estimates of free plasma concentration
[^18^F]FEMPA	N-{2-[2-^18^F-Fluoroethoxy]−5-methoxybenzyl}-N-[2-(4-methoxyphenoxy)pyridine-3-yl]acetamide	
[^18^F]DPA-714	N,N-Diethyl-2-(2-(4-(2-(^18^F)fluoroethoxy)phenyl)−5,7-dimethylpyrazolo[1,5-a]pyrimidin-3-yl)acetamide	
[^11^C]DPA-713	N,N-diethyl-2-(2-(4-methoxyphenyl)−5,7-dimethylpyrazolo[1,5-a]pyrimidin-3-yl)-acetamide	
[^18^F]FEPPA	[^18^F]-N-(2-(2-fluoroethoxy)benzyl)-N-(4-phenoxypyridin-3-yl) acetamide	
[^18^F]FEDAA1106	*N*-(5-fluoro-2-phenoxyphenyl)-*N*-(2-[^18^F]fluoroethyl-5-methoxybenzyl)acetamide	
[^18^F]PBR111	2-(6-chloro-2-(4-(3-fluoropropoxy)phenyl)imidazo[1,2-a]pyridin-3-yl)-N,N-diethylacetamide	
[^18^F]PBR06	[F-18]PBR06 [N-(2,5-dimethoxybenzyl)−2-(18)F-fluoro-N-(2-phenoxyphenyl)acetamide]	
Third Generation Tracers		
[^11^C]ER176	^11^C-(*R*)-*N*-*sec*-butyl-4-(2-chlorophenyl)-*N*-methylquinazoline-2-carboxamide	As compared to second generation ligands [18]: - High affinity for TSPO - Can reliably quantify uptake in all three binding affinity groups Challenges in Quantification: - Still requires binding affinity analysis - No suitable reference region Commentary for [^18^F]GE-180 [19]: - Although [^18^F]GE-180 has been deemed advantageous, publications indicate low brain uptake, almost flat tissue time-activity curves, difficulty in kinetic modeling, and poorly defined outcome parameters and generally suggest using other well-validated TSPO tracers
[^18^F]GE-180	(S)-N,N-diethyl-9-(2-[^18^F]fluoroethyl)−5-methoxy-2,3,4,9-tetrahydro-1H-carbazole-4-carboxamide	

Summary of all TSPO radiotracers used in the studies reviewed. Tracers have been sorted based on their generation, with notes on the limitations of each generation of radiotracer

*SNR* signal to noise ratio, *BP*_*ND*_ non-displaceable binding affinity, *TSPO* 18 kDA translocator protiein, *BBB* blood brain barrier

### DCE-MRI imaging

The BBB regulates nutrient exchange while limiting entry of neurotoxic compounds, maintaining a highly controlled CNS microenvironment and promoting proper brain clearance. The CNS, BBB, and immune system interact in a dynamic way such that inflammatory processes influence BBB function which in effect contributes to CNS function in health and disease [20]. When compromised, the BBB becomes hyperpermeable increasing glial activation, accumulation of neurotoxic blood-derived proteins, release of pro-inflammatory cytokines and resulting in neuronal dysfunction [4, 20].

DCE-MRI measures BBB permeability (BBBp) in vivo non-invasively (Table 3). DCE-MRI quantifies the difference in gadolinium (Gd)-based contrast agent concentration between blood plasma and the brain interstitial space over time [4]. Compared to other measures of BBB function such as direct examination of post-mortem tissue and the cerebrospinal fluid (CSF) albumin index (Q_alb_), DCE-MRI is less invasive, has superior spatial resolution, and allows the detection of early and subtle changes in BBBp across specific regions of interest. In addition, whereas conventional contrast-enhanced MRI only highlights a snapshot of the tissue at a singular point following contrast injection, DCE-MRI captures blood flow and permeability over time. By adding a temporal dimension, DCE-MRI offers BBB-related pharmacokinetic quantitative biomarkers of disease progression and treatment response and improves tissue characterization and diagnostic accuracy. Table 4 reviews TSPO-PET and DCE-MRI outcome measures. See Supplementary Table S2, Additional File 1 for a summary of which outcomes were reported in each of the reviewed studies.

**Table 3:** DCE-MRI review of quantification methods

BBB disruption enables the extravasation of low-molecular weight MRI contrast agents. In the DCE-MRI procedure, the combination of an intravenous injection of contrast agent followed by repeated acquisition of T1-weighted images permits measurement of signal enhancement as a function of time. This is because the accumulation of contrast material in the extravascular extracellular space (ESS) of the impacted tissue results in increased longitudinal relaxation rate and therefore increased signal intensity in T1-weighted images [4]. An appropriate pharmacokinetic model accounting for intrinsic tissue and acquisition parameters is necessary to relate the measured signal enhancement to the contrast agent concentration [21]. Numerous pharmacokinetic models have been reviewed previously in detail [22]. Briefly, models describe the exchange of contrast agent between the blood plasma and the EES. Target parameters include the fractional plasma volume (v_p_), fractional interstitial volume (v_e_), plasma flow (F_p_), permeability-surface area product (PS), and the volume transfer constant (K_trans_). Whereas the PS reflects the flow of contrast agent across the BBB normalized to the tissue volume and plasma concentration, Ktrans is the flux of contrast clearance normalized to the arterial plasma concentration. Ktrans is thereby contingent on the F_p_ supplying the capillaries as well as the PS [4] Most models require an arterial input function (AIF) which can be determined using a standard function, experimentally derived from a population-averaged AIF, or by deriving an arterial or vascular input function from a feeding artery or vessel proximal to the tissue of interest or one that is more global such as the internal carotid artery or superior sagittal sinus [4]. With the AIF known, one- or two-tissue compartment models can derive tracer concentration over time. The conventional Tofts model is a one-compartment model for weakly vascularized tissue as it assumes a negligible blood volume and K_trans_ [23]. Whereas the modified Tofts model includes a non-negligible blood compartment, thereby differentiating between signal enhancement due to contrast leakage from intravascular contrast [24]. Unlike the modified Tofts model, the Patlak model also describes a highly perfused two-compartment tissue but considers transport of contrast agent across the BBB to be unidirectional [25]	An example DCE-MRI acquisition and processing pipeline is depicted below using data from a temporal lobe epilepsy patient with a lesion in the left superior temporal gyrus (marked by orange arrow). A.) DCE-MRI acquisition with contrast injection. B.) Visualization of contrast agent uptake over time including before injection, at peak injection time, and towards the end of the acquisition period.C.) With the C(t) and AIF known, the Extended Tofts model was applied to derive parameter estimates of BBBp showing increased Ktrans in the lesion and peri-lesional areas

Overview of common DCE-MRI acquisition and quantification methods

**Table 4:** TSPO-PET and DCE-MRI outcomes

TSPO-PET measures	
**Outcome**	**Definition**
Total Distribution Volume (V_T_)	How much of the tracer is distributed in the tissue relative to its concentration in the blood [26] Studies also commonly report V_T_/*f*_p_ which is the total distribution volume corrected by the plasma free fraction
Distribution volume ratio (DVR)	The total distribution volume expressed as a ratio to the total distribution volume of a reference region [13]
Binding Potential (BP_ND_)	The ratio of specifically bound tracer to the nondisplaceable radioligand in the tissue [26]
Standard uptake value ratio (SUVR)	The concentration of radiotracer in the tissue in relation to the injected dose and body weight expressed as a ratio to the concentration of uptake in a reference region
Rate constants (K1, k2, k3, k4, an kb)	Parameters which describe the rate of tracer entry, retention, and removal between the plasma and tissue (K1) and between the tissue compartments (k2, k3, k4), derived from the two-tissue compartment model (2TCM) [11] In the 2TCM with an additional, irreversible compartment accounting for endothelial TSPO binding (2TC-1K), k_b_ is defined as the rate constant from plasma to the vascular compartment [27]
DCE-MRI Measures	
Volume transfer constant (K_trans_)	Rate of contrast agent flux between the arterial blood plasma and brain tissue extravascular extracellular space (EES) [24]
Leakage Rate (K_i_)	Rate of contrast agent flux between the arterial blood plasma (assuming a constant concentration) and brain tissue EES divided by the plasma concentration of the contrast agent defined by the Patlak model
Rate transfer constant (K_EP_)	The rate of back flux form brain EES to plasma defined as the ratio of K_trans_/v_e_, defined by the Tofts model [24, 25]
Leakage Volume (V_L_)	The fraction of the brain tissue where leakage is occurring which is calculated as the area under the leakage curve derived using the Patlak model [28]
Fractional Plasma Volume (v_p_)	The volume of plasma within the tissue [4]
Fractional interstitial volume (v_e_)	Volume of EES per unit of tissue volume [4]
Permeability Surface Product (PS)	The rate of exchange between the intravascular plasma volume and interstitial fluid volume [4]
EES fractional volume (Vb)	Whole blood volume per unit of tissue volume [24]
Total Permeability	The summed permeability across all voxels exceeding a defined threshold (e.g., 0.003/min) within regions of active BBB leakage, as derived from voxel-wise Patlak model analysis [29] Or, calculated as the voxel-wise normalized slope of contrast agent accumulation [30]

Definitions of TSPO-PET and DCE-MRI outcome measures

## Methods

**Fig. 1:**
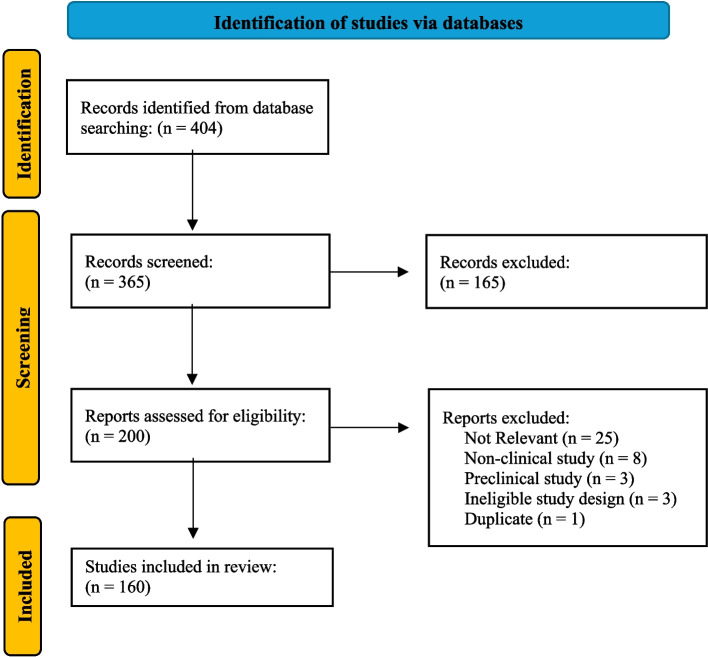
Study Selection criteria flow chart. PRIMSA-style flow diagram showing the number of records identified, screened, excluded, and included in the present review. This work is licensed under CC BY 4.0

We conducted a structured literature search to identify clinical studies of TSPO-PET and DCE-MRI in human subjects with AD, PD, epilepsy, MS, HD, Scz and depression. Two reviewers (ZK, ET) performed a search in PubMed (365 records) in January 2025 using search strings summarized in Supplementary Table S3 [see Additional File 1]. Given the clinical angle of this review, we excluded studies without human participants, methodological studies, abstracts, case reports, and systematic reviews. We applied a language filter (English) and limited the publication date to January 1, 2025. After exclusion of non-relevant studies 200 publications remained, of which 160 were original publications included for this review. References of all publications were checked for additional relevant investigations. The search was last performed on July 1 st, 2025. 22 additional relevant studies had been published. The PubMed literature search is described based on the PRISMA flowchart [31] (Fig. 1).

### TSPO-PET and AD

In AD, the interaction of misfolded and aggregated proteins with glial cells releases inflammatory mediators and upregulates clearance of pathological proteins [32]. However, large increases of pro-inflammatory molecules can lead to excessive neurotoxicity which in turn prompts additional glial activation. The bidirectional relationship between glia and protein aggregation and clearance is a complex and dynamic contribution to AD pathology [33]. Although mounting evidence indicates that this chronic neuroinflammatory response is a vital component of AD pathology, whether it is protective, toxic, or both across disease progression remains controversial.

Both early and later progressive stages of AD have been associated with increased TSPO uptake in cortical regions implicated in AD pathology including parietal, hippocampus (HIPP) and other medial temporal lobe (MTL) regions [13, 34, 35]. Glial activity may also be contingent on age of disease onset—early-onset AD (EOAD) patients exhibit greater [^11^C]PBR28 binding than late-onset (LOAD) patients [36] and rapid longitudinal increases in global [^11^C]PK11195 binding potential (BP) occur in parallel with more severe cognitive deterioration in EOAD as compared to LOAD [37]. Early glial activation correlates with later clinical deterioration, making it an attractive candidate as a disease biomarker.

AD-related metabolic risk factors including body mass index (BMI), insulin resistance, and high-sensitivity C-reactive protein (CRP), a measure of peripheral inflammation have been associated with TSPO uptake in cognitively unimpaired elderly with and without amyloid (Aβ) deposition [38, 39]. Amyloid-positive (Aβ +) controls have increased [^18^F]DPA-714 SUVR compared to those without Aβ pathology [40]. Age dependent increases in Aβ deposition and TSPO uptake have also been identified in healthy controls (HCs) [41]. Metabolic risk factors in aging populations may contribute to Aβ accumulation via increases in glial activation.

Cortical Aβ load has also been linked to glial activation across AD progression, with ligand uptake varying by risk group and disease stage and associating with CSF Aβ markers. (Table S4) Co-localization of glial activation and Aβ aggregates in cortical and limbic regions increases with disease progression, [37, 40, 42–44] supporting a role for glia in Aβ clearance [42]. On the contrary, some studies report no associations between glial activity and Aβ deposition [36, 38, 45]. Glial activity levels have also been shown to overlap between prodromal, symptomatic, and HCs groups [40, 46]. One study identified a sex effect on TSPO signal independent of Aβ pathology [47]. These studies underscore the heterogeneity of glial activity in AD [48], which may translate clinically to different rates of disease progression and sex-specific outcomes.

TSPO uptake is also elevated across all Braak stage regions in mild cognitive impairment (MCI) and AD patients relative to HCs [35, 49] and correlates with tau pathology in HCs and individuals across the AD continuum, regardless of Aβ status [35, 42]. Glial activity may contribute to tau spread and neurodegeneration [49] through a positive feedback loop involving pro-inflammatory products of glial activation and tau hyperphosphorylation [42]. Studies suggest that tau pathology is more closely linked to glial activity than Aβ [35, 42] and may drive the relationship between glial activation and Aβ pathology [44]. However, some studies report no correlations between inflammation and tau pathology, [43] potentially reflecting the differing temporal trajectories of Aβ deposition, glial activation, and tau aggregation, with glial involvement occurring early during Aβ plaque formation, preceding tau pathology [43]. Further, associations between glial activation and functional and cognitive decline also remain inconsistent across disease stages and in Aβ + individuals (See Supplementary Table S4, Additional File 1).

While studies evidence incremental increases of TSPO expression across disease progression, [36] Fan and colleagues propose a biphasic model wherein glial activity in prodromal AD is initially protective, while later-stage activation is neurotoxic [32]. Higher early TSPO uptake has been associated with slower cognitive decline, [40, 46] while reduced TSPO signal in cognitively normal Triggering Receptor Expressed on Myeloid cells 2 carriers suggests impaired early microglial protection may elevate AD risk [50]. Moreover, peaks in glial activity appear to follow the temporal order of Aβ and tau pathology, expanding the biphasic model. Early protective glial activity may facilitate Aβ clearance [32, 46] whereas later glial activity aligns with tau-mediated neurodegeneration, potentially independent of Aβ pathology [36, 44, 46].

Understanding the temporal dynamics of glial activation in AD requires accounting for distinct microglial phenotypes, [46] as their density, morphology, and behavior vary with the duration and severity of pathology [51]. The roles of microglia do not distinctly coincide with disease stage due to individual differences in genetic risk, pathological burden, and clinical presentation. This variability underscores the need for individualized treatment paradigms if one is to consider the translation of these findings to interventional studies aimed at modifying glial activation [44].

### DCE-MRI and AD

Neurovascular unit (NVU) damage can initiate a complex cascade leading to BBB disruption and cerebral microvasculature changes that contribute to AD pathology. BBB dysfunction is evident in early AD stages [52]. The HIPP and its associated subregions harbor increases in BBBp in older vs. younger HCs without cognitive impairment, [53] and in MCI and early AD compared to HCs, [54] despite no differences in structural volume between groups [52]. In older HCs, age-related increases in BBBp were attenuated by white matter hyperintensities (WMH) volume and cortical thickness, which are typically observed in normal aging. However, these increases in BBBp remained significant in the context of HIPP volume, [55] suggesting that MTL BBBp may reflect AD-related pathology beyond normal aging, a pattern not observed in impaired individuals [52, 54, 56]. These findings indicate that early progressive changes to BBBp may precede the onset of AD and worsen with disease progression.

Although BBBp changes have been linked to AD, it remains unclear whether BBB breakdown results directly from AD pathology or from underlying NVU dysfunction. Associations between BBBp and WMH in cohorts of HCs, MCI, AD [57] and vascular MCI patients, [58] suggest a stronger association to microvascular dysfunction than neurodegeneration. Vascular risk burden contributes to both BBBp and white matter (WM) injury, which correlates with cognitive dysfunction in AD [29]. NVU impairment may also reduce cerebral blood flow, promoting early global vascular damage [28]. Associations between BBBp changes and grey matter (GM) [52] and WM regions [59] persist even when controlling for vascular comorbidities and without associated WMH, [54, 59] implying that BBB impairment stems in-part from underlying AD pathology rather than just concurrent cerebrovascular disease.

#### BBB dysfunction, Aβ and Tau

The link between BBB dysfunction, abnormal Aβ and tau protein accumulation is not well understood. Studies in HCs and MCI patients found no differences in MTL K_trans_ as a function of Aβ or tau pathology [52–54, 56, 60]. Increased BBBp in the HIPP, hippocampal subfields, and parahippocampal gyrus could predict cognitive impairment independent of CSF Aβ_1–42_ and phosphorylated tau, [52] but cortical K_trans_ does not change as a function of Aβ PET or cognitive score in MCI and dementia patients [61]. Although BBB breakdown may serve as an early, independent biomarker of cognitive decline [52, 57–59] (See Supplementary Table S5, Additional File 1), its role in the context of Aβ burden remains ambiguous. Higher cortical K_trans_ was associated with decreased Aβ and tau in Aβ + individuals, but positively related to CSF total tau protein in Aβ– individuals, suggesting that cortical BBBp may reflect neuronal injury regardless of Aβ burden [61]. This dual relationship highlights the BBB’s role in both protecting against and clearing toxic proteins, paralleling the biphasic nature of glial activation in AD. While early BBB dysfunction may impact cognitive decline independently of Aβ or tau, later breakdown may exacerbate neurodegeneration.

#### BBB dysfunction and APOE4

Apolipoprotein (APOE4) has also been implicated in increased BBB dysfunction and brain capillary pericyte degeneration [60]. Some studies report no significant genotype-dependent differences in BBBp in disease free APOEε4 (ε3/ε4 and ε4/ε4) and APOEε3 carriers (ε3/ε3) [62] as well as in a mixed cohort of HCs, MCI, and AD patients [54]. While other studies showed increased cortical and HIPP BBBp in healthy and impaired APOEε4 carriers independent of Aβ or tau pathology [53, 56, 60]. In fact, BBBp in MTL regions and entorhinal cortex correlates with cognitive impairment in APOEε4 carriers, irrespective of AD-related pathology and entorhinal microstructural abnormalities, [56, 60] suggesting that the interaction between BBB dysfunction and AD pathophysiology may accelerate regional neurodegeneration [56]. Although subtle changes in MTL BBB integrity occur early in AD, genetic factors and Aβ pathology may heighten susceptibility to neurovascular dysfunction, exacerbating clinical progression.

#### BBB dysfunction and standardized biomarkers of BBB function

Imaging markers of BBBp in the HIPP and its subregions have been associated with standardized biomarkers of BBB function and integrity, including Q_alb_, CSF fibrinogen and plasminogen, and CSF soluble platelet-derived growth factor receptor β (sPDGFRβ), a marker of BBB-associated pericytes key to maintaining BBB integrity, in APOE4 carriers and MCI patients [52]. However, one study found no association between sPDGFRβ or Qalb and HIPP K_trans_ in a HC, MCI, and AD cohort [54]. These differing outcomes highlight the potential divergence of central and peripheral immune activation in AD which may not align temporally across disease stages.

### TSPO-PET and PD

Although increased [^18^F]DPA-714 uptake in the substantia nigra (SN), pons, putamen, thalamus, and fronto-temporal cortical regions has been reported in PD patients relative to HCs, [63, 64] other studies have not replicated these findings or have not linked glial activation to clinical severity or putaminal dopaminergic terminal density measured via Dopamine Transporter (DAT) uptake [65, 66]. [^18^F]DPA-714 uptake in nigrostriatal regions correlated with several CSF-derived chemokine measurements in early PD [64]. In a PD clinical trial, [^11^C]PBR28 uptake decreased in nigrostriatal regions following anti-inflammatory treatment- but these decreases did not correlate with dopaminergic innervation as measured by [^18^F]FE-PE2I [67]. However, another study using [^11^C]PBR28 failed to detect glial activity in PD patients, despite the co-occurrence of high levels of neuroinflammation marker CSF chitinase 3-like 1 protein [68]. Since neuronal loss in PD starts in the ventral tier of the substantia nigra pars compacta (SNpc) projecting to the dorsal putamen it is possible that increased TSPO binding may parallel dopaminergic degeneration only in the early stages of PD, preceding the apoptosis of dopaminergic SNpc neurons.

### DCE-MRI and PD

There is a growing body of evidence suggesting that BBBp may play a causative role in the degeneration of SNpc dopaminergic neurons [69]. To our knowledge, there is only one recent clinical study of PD investigating regional alterations in BBBp using DCE-MRI. The SN, normal appearing white matter (NAWM), and posterior cortex had elevated K_trans_ in PD patients compared to HCs [69]. Posterior cortical regions in PD also displayed hypo-perfusion, strengthening the link between BBB disruption and perfusion deficits in these regions [69].

### TSPO-PET and epilepsy

Brain inflammation has been cited as both a cause and consequence of epilepsy. Activated microglia, reactive astrocytes, and pro-inflammatory agents hold reciprocal roles in the hyperexcitability observed in seizure foci and their networks [70]. Evidence for neuroinflammatory mechanisms of epilepsy has stemmed from murine models, with limited data available from in vivo studies in human subjects [71].

Elevated TSPO uptake has been observed in regions proximal and distant to the epileptogenic zone (EZ) and epileptogenic lesions across several epilepsy etiologies. Studies report increased TSPO uptake asymmetry in ictal-onset regions in drug-resistant epilepsy (DRE) patients with seizure foci outside of the MTL [72] and in temporal lobe epilepsy (TLE) patients relative to HCs [70]. HIPP uptake asymmetry was greater in TLE patients with mesial temporal sclerosis than in those with nonspecific pathology [70]. Additionally, increases in [^11^C]DPA713 SUVR were shown in lesional regions of interest (ROIs) as compared to non-lesional ROIs in intractable child-onset focal epilepsy, particularly in cases of gliosis, cortical malformation, [73] and tuberous sclerosis [74]. TSPO-PET detects inflammation beyond hypometabolic regions identified by FDG-PET [75]. TSPO activity also extends beyond seizure foci and lesion boundaries in both temporal and extratemporal regions [70, 74]. Although one study found increased binding ipsilateral to the EZ, most patients demonstrated bilateral uptake [72]. Importantly, seizure frequency, time since last seizure, or epilepsy duration do not appear to impact TSPO uptake [70, 72, 73].

TSPO-PET may also be informative in acute symptomatic seizures related to autoimmune encephalitis. The majority (90%) of patients with autoimmune encephalitis (AIE) mediated by antibodies against leucine-rich glioma-inactivated 1 not only showed increased [^18^F]DPA-714 SUVR in limbic regions, corresponding to structural MRI lesions, but also in the caudate nucleus and frontal cortex [76]. The type of seizure (tonic–clonic versus faciobrachial dystonic seizures) correlated with different regional distribution of increased TSPO uptake.

Inter-individual variability in binding values [70, 72] and regional inconsistencies in TSPO uptake [74] represent technical challenges in the clinical adoption of TSPO-PET in epilepsy imaging standard of care.

### DCE-MRI and epilepsy

Inflammation may also contribute to seizures via BBB dysfunction [70]. Studies examining in vivo BBB dysfunction in epilepsy are scarce.

Patients with both generalized and focal DRE both have greater BBB dysfunction relative to HCs, primarily localized in frontal and temporal cortical GM regions [30]. For those with MR-positive lesions, BBBp was identified ipsilaterally and contralaterally to lesion location [30]. Changes in BBBp are also reported around cysticercal lesion associated with Neurocysticercosis, the leading cause of epilepsy worldwide. The greatest BBBp occurs at the colloidal stage which is associated with intense perilesional inflammation, increased BBB breakdown, elevated serum levels of matrix metalloproteinase (MMP-9), and the development of seizures [77]. High baseline MMP-9 and K_ep_ values predicted seizure recurrence [78].

BBB dysfunction has also been implicated in the development of status epilepticus. Patients with new onset refractory status epilepticus had significantly increased K_trans_ in the HIPP, basal ganglia (BG), and a trend in the thalamus, while AIE patients had significantly increased K_trans_ in the periventricular WM and a trend in the HIPP as compared to HCs, despite normal structural MRI, suggesting that changes in BBB integrity are independent of changes in structural integrity [79].

### TSPO-PET and MS

TPSO-PET has been extensively utilized as a tool to measure glial activation in MS and may have utility in differentiating MS subtypes and detecting both diffuse and compartmentalized glial activity [80, 81].

Quantitative Susceptibility Mapping, which is typically used to classify the iron-rich dense rim of activated microglia characteristic of smoldering lesions as rim + (active) or rim- (inactive), has been validated against TSPO-PET [82, 83]. Rim + lesions exhibit increased TSPO uptake compared to inactive lesions [82, 84]. Furthermore, post-mortem analysis of brain tissue from MS patients demonstrated that [^11^C]PK11195 uptake co-localizes with iron-containing CD68+ microglia and macrophages [82]. A higher number of active lesions identified by TSPO uptake also correlated with worsening disability and predicted MS disease progression [83, 85, 86].

TSPO radioligands can also capture glial activity beyond focal lesions, as MS pathology includes widespread inflammation below the threshold of lesion formation. Studies utilizing [^11^C]PK11195 and [^11^C]PBR28 have demonstrated increased whole brain tracer uptake in MS patients compared to HCs [81, 87–89]. Moreover, elevated uptake in the NAWM is well documented, particularly in secondary progressive MS (SPMS), where diffuse and low-grade neuroinflammation is more widespread [87, 89, 90]. NAWM uptake predicts disease progression, [84] is higher in SPMS than relapse-remitting MS (RRMS), and correlates with WM microstructural damage and clinical disability [91]. In RRMS, higher lesion load predicts later NAWM [^11^C]PK11195 DVR increases [81]. Moreover, both [^11^C]PK11195 and [^18^F]PBR06 NAWM uptake have been used as biomarkers in clinical trials, with reductions following treatment with natalizumab, rituximab, or fingolimod- agents targeting microglial cells [85, 92, 93]. However, NAWM [^18^F]PBR06 uptake does not normalize after treatment, indicating persistent inflammation from smoldering lesions [85]. These findings implicate diffuse inflammation as a mechanism by which smoldering lesions promote demyelination [87].

TSPO-PET has also revealed immune activation beyond WM, notably in the thalamus and choroid plexus (CP). Thalamic uptake of [^11^C]PK11195 and [^11^C]PBR28 predicts disease progression across MS subtypes, [81, 88] with increased [^11^C]PBR28 DVR in MS patients relative to HCs WM and linked to increased cortical thinning [94]. [^11^C]PBR28 thalamic uptake was greater in SPMS than RRMS [89]. Similarly, [^18^F]DPA-714 uptake is elevated in periventricular regions, decreasing with distance from the ventricles and correlating with microstructural damage [95]. Also, CP [^18^F]DPA-714 uptake was higher in both pre-symptomatic and RRMS patients than HCs, [96, 97] suggesting that CP inflammation may be an early feature of MS [97]. Moreover, [^18^F]DPA-714 uptake co-localized with CD163 + macrophages in post-mortem analysis of CP tissue from MS patients [96].

Increased [^11^C]PBR28 uptake in the cortico-meningeal compartment, particularly in RRMS relative to SPMS, [90] suggests that meningeal inflammation contributes to demyelination [98]. Combined [^11^C]PBR28 PET and ex vivo immunohistochemistry revealed elevated TSPO expression on MHC-class II+ meningeal macrophages and cortical-activated TMEM119+ microglia, supporting a role for compartmentalized meningeal inflammation in MS-related demyelination [98].

Although several studies have failed to find differences in [^11^C]PK11195 uptake in MS patients compared to HCs [99, 100] second- and third-generation radioligands including [^18^F]PBR06, [^18^F]PBR111, and [^18^F]GE-180 have shown promise in tracking MS-related glial activation [80, 101, 102]. Additionally, male MS patients exhibited higher [^11^C]PK11195 uptake across several ROIs compared to females matched by disease status [103]. This sex-driven heterogeneity in TSPO binding may thereby contribute to worse disease outcomes inherent in male MS patients [103]. Future studies may benefit from multi-tracer PET approaches to simultaneously assess glial activation and myelin integrity in MS [100].

### DCE-MRI and MS

By disrupting BBB integrity, MS enables macrophages, along with other components of the immune system, to enter the brain parenchyma and form demyelinating lesions [104].

DCE-MRI is the diagnostic gold standard in MS for differentiating contrast-enhancing (CE) from non-contrast enhancing (NCE) lesions, with CE lesions indicating active inflammation and BBB disruption [104, 105]. CE lesions show higher K_trans_, K_i_, and V_p_ values as compared to NCE lesions and NAWM [2, 105]. Reports of higher K_i_ in NAWM of RRMS patients compared to WM from HCs, suggests that DCE-MRI can detect subtle BBB abnormalities typically identified only histologically [2, 106]. Although K_i_ has been validated as a marker of BBBp, its interpretation warrants caution, as it may reflect Gd entry via the CP into the CSF rather than true BBBp [2, 106].

DCE-MRI has also shown promise as a tool for monitoring treatment response in MS. Patients treated with anti-inflammatory drugs who maintain No Evidence of Disease Activity-3 (NEDA-3) status display reduced K_i_ in NAWM and GM after treatment [107, 108]. MS patients who maintain NEDA status display higher K_i_ at baseline relative to the patients for whom treatment was ineffective [107]. Conversely, following treatment, patients with persistent disease had higher NAWM K_i_ compared to those who had no evidence of active disease [108]. As anti-inflammatory therapies like alemtuzumab, natalizumab, and fingolimod target luminal drivers of MS (peripheral inflammation which can then affect brain immunity), treatment efficacy may depend on BBB integrity. Patients with impaired BBB may respond better, while those with abluminal driven disease—less accessible to these agents—may exhibit subtle inflammation without lesion formation or continue to develop lesions. Thus, K_i_ may help predict suboptimal responses to disease-modifying therapies.

### TSPO-PET and HD

Mutant huntingtin causes dysfunction and degeneration of striatal medium spiny neurons and cortical neurons, driving the motor, cognitive, and psychiatric symptoms of HD through mechanisms involving oxidative stress and immune activation [109]. While most evidence of gliosis in HD comes from postmortem studies, limited in vivo data suggest that glial activation may contribute to HD pathogenesis [109].

Increased TSPO expression was identified in premanifest HD, [109] early symptomatic HD, [110] and manifest HD gene carriers [111] in BG structures including the globus pallidus and putamen, and in the caudate [112] relative to HCs. In premanifest HD gene carriers, increased peripheral markers of inflammation (IL-1β, IL-6, IL-8 and tumor necrosis factor alpha (TNF-α) produced by stimulated peripheral blood monocytes) correlated with elevated TSPO expression in somatosensory cortex [109]. Among premanifest and manifest HD gene carriers, increased [^11^C]ER176 SUVR in bilateral putamen and pallidum was associated with disease progression and increased atrophy of both regions, demonstrating that glial activation contributes to neuronal degeneration [111]. Further, whereas one study reported associations between [^11^C]PBR28 DVR and CAG repeat length and disease duration, no associations were found with age or clinical severity in patients carrying 40–49 CAG repeats [112]. Therefore, TSPO-PET appears to inform HD progression in relation to genetic risk, disease duration, and neurodegeneration.

One double-blind, placebo-controlled trial of laquinimod, an immunomodulator, found no significant change in [^11^C]PBR28 DVR in the caudate or putamen or clinical symptoms for HD receiving two different doses compared to placebo [112]. It is possible that glial activity is a broad target that does not respond to immunomodulation in HD, or that TSPO-PET lacks the sensitivity to detect subtle changes in glial activity.

### DCE-MRI and HD

To our knowledge, there are no studies available looking at BBBp using DCE-MRI in HD populations.

### TSPO-PET and schizophrenia

Neuroinflammation may contribute to brain morphometric changes in Scz [113]. Studies have established modest increases in whole-brain GM tracer uptake in stable Scz patients as compared to HCs [114, 115]. Progressive GM volume loss in Scz may result from glial-mediated disruptions in synaptic pruning, as increased GM [^11^C]PBR28 uptake corresponds to cortical GM volume reduction [113].

Focal immune activation may also be a feature of psychosis. HIPP [^11^C]PK11195 uptake decreased in Scz patients who were treated with valacyclovir, [114] administered based on the hypothesis of a viral trigger for psychosis, but was not associated with disease severity [114]. Therefore, although glial activity may be present in the HIPP of Scz patients, it may not be a viable marker of clinical severity or treatment response. In fact, several studies challenge the clinical utility of TSPO-PET in Scz as Scz patients did not differ from HCs in [^11^C]DPA713 uptake [116] despite the elevation of peripheral immune markers (plasma & CSF IL-6), [117] while other studies found that age and antipsychotic medication use, but not disease status, correlated with increased [^11^C]PK11195 uptake [118].

### DCE-MRI and schizophrenia

To our knowledge, there has been one study which demonstrated that Scz patients harbored elevated bilateral thalamic K_trans_ compared to HCs, correlating with illness duration and severity [119].

### TSPO-PET and depression

Elevated TSPO uptake has been reported in brain regions linked to depression including the anterior cingulate cortex (ACC), insula, prefrontal cortex (PFC), parahippocampus, ventral striatum and dorsal putamen in patients with depression, [120, 121] post-COVID depressive symptoms, [122] and late-life depression [123] relative to HCs. In MS patients, elevated HIPP [^18^F]PBR111 V_T_ was observed in those with a major depressive episode (MDE) compared to those without or recent but resolved MDE. [124] Elevated TSPO uptake has also been associated with suicidal thinking, [125] stress-related suicidal ideation severity, [126] and cognitive dysfunction [127]. Depression as a primary psychiatric disorder and comorbidity is associated with immune activation, but studies have not determined whether glial activity is a mediator of the comorbidity.

Medication-naïve MDE patients have higher TSPO uptake than both HCs [125, 128] and medicated patients with major depressive disorder (MDD) [129]. A longer duration of untreated MDD has been linked to increased TSPO signal [130]. In fact, one study found no difference in [^11^C]PBR28 V_T_ between medicated MDD patients and HCs, despite similar depression scores across groups, indicating that antidepressants may normalize TSPO expression even without resolving depressive symptoms [129]. An open-label trial of celecoxib, a cyclooxygenase-2 inhibitor, showed symptom improvement only in patients with high baseline [^18^F]FEPPA V_T _ [131]. Conversely, a trial of minocycline found no reductions in [^18^F]FEPPA V_T_ or association between change in uptake and depressive symptoms [132]. In drug-naïve MDD patients undergoing cognitive behavior therapy, [^18^F]FEPPA V_T_ was significantly reduced and associated with an amelioration of depressive symptoms [133]. Both pharmaceutical [134] and cognitive-behavioral interventions [133] may regulate glial activity in depressed populations, and TSPO-PET may provide a surrogate marker of glial activity changes.

Although peripheral inflammation markers are reportedly increased in MDD, some studies have found no association between peripheral and central markers of inflammation [121]. While BMI is a known contributor to chronic low-grade inflammation and a potential confounder in TSPO studies, no link between BMI and TSPO signal was observed in depressed patients and HCs [120]. This may reflect a limited BMI range in study samples or suggest that peripheral-metabolic inflammation may not directly contribute to central inflammation in depressed populations. However, serum adiponectin, which exerts anti-inflammatory effects, negatively correlated with HIPP [^11^C]PK11195 BP_ND_ in MDD patients [128]. Moreover, studies have linked peripheral inflammation to brain TSPO signal. [^11^C]PBR28 V_T_ in the subgenual PFC and ACC correlated with CSF IL-5 [129] and serum prostaglandin E2, TNF-α, and [^18^F]FEPPA V_T_ in MDE and treatment-resistant MDD correlated with CRP levels [135]. Overall, while peripheral immune dysfunction is implicated in depression, its direct impact on glial activation remains unclear, suggesting that other factors may be involved.

### DCE-MRI and depression

To our knowledge, there are no available studies of BBBp using DCE-MRI in depressed populations.

## Summary

Immune activation involving glia and BBB dysfunction is a vital contributor to the pathophysiology of various neuropsychiatric disorders. Over the past decade, TSPO-PET and DCE-MRI have demonstrated increased clinical utility for tracking inflammatory mechanisms contributing to neurological disease onset and progression. These imaging tools may also serve as biomarkers of treatment response for therapies with direct or indirect anti-inflammatory mechanisms. Studies demonstrate both focal and widespread glial activity and BBB dysfunction underlying numerous pathologies. In conditions such as AD and HD, elevated immune activation is detectable in prodromal or early clinical stages, highlighting neuroinflammation as a potential target for intervention in at-risk populations.

Despite advances in second- and third-generation TSPO-PET tracers, methodological variability contributes to inconsistencies in study findings. Input function derivation methods differ between studies, with some using gold standard arterial sampling while others opt for less invasive or image-derived methods, as arterial sampling may impact recruitment in study populations with neurologic impairment [112] (Table 1). Additionally, the lack of standardized quantification methods has led to numerous modeling approaches, resulting in divergent parameter estimates and reported outcomes. (Table S2, Additional File 1) Suggestions for optimal quantification have been published [11].

Radiotracer selection also influences outcomes. Although second- and third-generation ligands offer improved SNR (Table 2) and are expected to enhance sensitivity to subtle immune changes, study findings remain inconsistent across tracers. This suggests that even optimized TSPO-PET may have limited sensitivity to detect subtle changes in glial activity.

In fact, TSPO-PET lacks the SNR and specificity to distinguish between potential contributions from different microglia and astrocyte subtypes. While microglial activation was previously categorized as proinflammatory M1-like and neuroprotective M2-like phenotypes, it is now recognized as a spectrum of functional differentiation patterns that PET imaging cannot discern [51]. Microglial heterogeneity is characterized by location, sex-specific cellular-origin, colonization patterns, morphology, and gene expression which translate to diverse phenotype and function [51]. This diversity is dynamic and contingent on the cellular environment, especially in pathological environments or regions with BBB disruption. For example, WM microglia differ phenotypically and functionally from those in GM and may contribute to WM abnormalities [51]. Across the CNS, microglia are thus expected to be fine tuning neuronal and glial functions and circuitry, influencing disease onset, progression and treatment response.

Interpreting TSPO-PET signal is further complicated by potential contributions from reactive astrogliosis, whose TSPO expression remains debated [8]. Similarly, TSPO signal may also stem from macrophages and endothelial cells, though evidence suggests their signal contributions do not significantly account for increased TSPO binding relative to microglia [8, 98]. Additionally, recent reports suggest that TSPO signal is driven by microglial burden, rather than increased TSPO expression per cell [10]. Therefore, increased glial activity may not translate to increased TSPO-uptake, as glial activation can occur independently of elevations in density [10]. The difference between activated state and density of cells may explain some of the discrepancies found between histopathological studies and TSPO-PET, for example in Parkinson's disease [10]. Moreover, both the cellular substrate and mechanism promoting elevated TSPO uptake may vary depending on the disease [8, 10].

Similarly, for DCE-MRI, methodological differences in data acquisition, signal modeling techniques, and input function derivation results in variable quantification of parameter estimates, complicating cross-study comparisons [4, 78] (Table 3 and Table S2, Additional File 1). While typically used to detect high BBB breakdown, DCE-MRI has also revealed subtle permeability changes close to intact values in MCI, AD, and MS. Notably, K_trans_ values in mildly impaired BBB are one to two orders of magnitude lower than in highly damaged regions, [78, 136] making accurate detection sensitive to factors such as acquisition time, temporal resolution, pharmacokinetic modeling approach and analysis software [136]. DCE-MRI may also fail to detect chronic inflammation, as BBB disruption occurs only for a short time during lesion formation [80]. This transient nature of BBB disruption should be a consideration when designing DCE-MRI studies especially for conditions such as epilepsy, in which seizures can temporarily impact the BBB. Lastly, existing vascular pathology can influence DCE-MRI findings and should be considered in studies of the BBB [29, 57, 58].

The relationship between peripheral and central immunity across disease states may also affect signal interpretation for both TSPO-PET and DCE-MRI. For instance, DCE-MRI is effective for detecting BBB dysfunction and leakage but is not well-suited for psychiatric conditions such as MDD wherein mild levels of peripheral cytokine production reduce BBBp [137], which may partly explain the lack of evidence in the literature. Reductions in BBBp also impact TSPO tracer extravasation across the BBB, with studies reporting an inverse association between TSPO tracer perfusion and peripheral inflammation, which further contributes to the mixed study findings in these disorders [138]. This may explain why studies using a reference region for quantification find increased TSPO expression whereas those utilizing AIF input report decreases.

## Future directions

PET and DCE-MRI outcome measures can overlap between patient groups, and variability in acquisition and quantification protocols limits the ability to define reliable cutoffs for distinguishing disease stages or symptom phenotypes. Moreover, reductions in BBBp are not currently detectable using DCE-MRI and may impact tracer delivery to the brain, further limiting study interpretation. This not only highlights the value of multimodal PET and DCE-MRI studies to complement established disease biomarkers (e.g. Aβ, tau, DAT-PET, cognitive decline, structural MRI, etc.), but also emphasizes the importance of studying BBB and inflammatory mechanisms in tandem. Co-localization studies [8, 82] are also needed to isolate the potential contributions of microglial phenotypes, astrocytes, and BBB integrity to neuroinflammation. In addition, while factors such as age, sex, and BMI are known to influence imaging metrics, [47, 103, 118] given the wide range of acquisition and analysis techniques, future studies should consider characterizing the impact of these variables on their chosen methods within study populations and HCs. Standardizing acquisition and analysis protocols will improve sensitivity to early immune and BBB changes and enhance cross-study comparability. In fact, multi-site PET and DCE-MRI studies with harmonized methods would enhance reproducibility and promote clinical utility. Finally, given that distinct inflammatory profiles are dependent on a complex interplay between cell types, existing markers of pathology, genetics, clinical risk factors, and disease stage and duration, future multi-modal and co-localization studies should be conducted in longitudinal cohorts to better track disease progression and outcomes.

## Supplementary Information

Supplementary Material 1.

## Data Availability

The dataset used in the current study is not publicly available due to restrictions related to the ongoing NIH-funded research project, which has not yet been completed, but it may be made available from the corresponding author upon reasonable request.

## Abbreviations

BBB: Blood brain barrier
CNS: Central nervous system
AD: Alzheimer’s disease
PD: Parkinson’s disease
MS: Multiple Sclerosis
HD: Huntington’s disease
Scz: Schizophrenia
TSPO: Translocator protein
PET: Positron Emission Tomography
DCE-MRI: Dynamic Contrast-Enhanced Magnetic Resonance Imaging
SNR: Signal-to-noise ratio
HAB: High-affinity Binders
MAB: Mixed-affinity Binders
BP_ND_: Non-displaceable Binding Potential
BBBp: BBB Permeability
Gd: Gadolinium
CSF: Cerebrospinal fluid
Q_alb_: CSF-albumin Index
V_T_: Total Distribution Volume
DVR: Distribution Volume Ratio
SUVR: Standard Uptake Value Ratio
2TCM: Two-tisseue Compartment Model
2TC-1K: 2TCM accounting for irreversible endothelial TSPO binding
K_trans_: Volume Transfer Constant
EES: Extravascular extracellular space
K_i_: Leakage Rate
K_ep_: Rate Transfer Constant
V_L_: Leakage Volume
V_p_: Fractional Plasma Volume
v_e_: Fractional interstitial volume
PS: Permeability Surface Product
Vb: EES fractional volume
HIPP: Hippocampus
MTL: Medial Temporal Lobe
EOAD: Early-onset Alzheimer’s disease
LOAD: Late-onset Alzheimer’s disease
BP: Binding Potential
BMI: Body Mass Index
CRP: C-reactive protein
Aβ: Amyloid
Aβ +: Amyloid-positive
HCs: Healthy Controls
MCI: Mild Cognitive Impairment
NVU: Neurovascular Unit
WMH: White Matter Hyperintensities
WM: White Matter
GM: Grey Matter
APOE4: Apolipoprotein 4
sPDGFRβ: Soluble Platelet-derived Growth Factor Receptor β
SN: Substantia Nigra
DAT: Dopamine Transporter
SNpc: Substantia Nigra Pars Compacta
NAWM: Normal Appearing White Matter
EZ: Epileptogenic Zone
DRE: Drug Resistance Epilepsy
TLE: Temporal Lobe Epilepsy
ROIs: Regions of Interest
AIE: Autoimmune Encephalitis
MMP-9: Matrix Metalloproteinase
BG: Basal Ganglia
SPMS: Secondary Progressive Multiple Sclerosis
RRMS: Relapse-remitting Multiple Sclerosis
CP: Choroid Plexus
CE: Contrast Enhancing
NCE: Non-contrast Enhancing
NEDA-3: No Evidence of Disease Activity-3
ACC: Anterior Cingulate Cortex
PFC: Prefrontal Cortex
MDE: Major Depressive Episode
MDD: Major Depressive Disorder
TNF-α: Tumor Necrosis Factor Alpha
