# Supplementary material for: Imaging brain inflammation and blood brain barrier permeability in neurological and psychiatric diseases: a review
Source: J Neuroinflammation. 2025 Nov 19;22:275. doi: 10.1186/s12974-025-03598-x (PMC12629060; doi:10.1186/s12974-025-03598-x)
Supplement: Supplementary file 1 — Supplementary Material 1. [file 12974_2025_3598_MOESM1_ESM.docx]

**SUPPLEMENTARY MATERIALS**

**Table S1. TSPO Radiotracers used in Reviewed Studies**

Summary of all TSPO radiotracers used in the studies reviewed. Tracers have been sorted based on their generation. Reviewed studies have been organized based on disease and tracer used.

| **Tracer Name** | | **Disease Condition** | | | | | | |
| --- | --- | --- | --- | --- | --- | --- | --- | --- |
| **First Generation Tracers** | | | | | | | | |
| **Short Name** | **Long Name** | **AD** | **PD** | **Epilepsy** | **MS** | **HD** | **Scz** | **Depression** |
| [^11^C]-PK11195 | 1-(2-chlorophenyl)-N-[11C]methyl-N-(1-methylpropyl)-3-isoquinoline carboxamide | [1–18] | [19,20] |  | [21–34] | [35] | [36–39] | [40–43] |
| **Second Generation Tracers** | | | | | | | | |
| [^11^C]-PBR28 | N-((2-(methoxy-^11^C)-phenyl)methyl)-N-(4-phenoxy-3-pyridinyl)acetamide | [15,44–54] | [55–58] | [59,60] | [61–66] | [67,68] | [69–71] | [72,73] |
|  |  |  |  |  |  |  |  |  |
| [^18^F]-FEMPA | N-{2-[2-^18^F-Fluoroethoxy]-5-methoxybenzyl}-N-[2-(4-methoxyphenoxy)pyridine-3-yl]acetamide | [74] |  |  |  |  |  |  |
| [^18^F]-DPA-714 | N,N-Diethyl-2-(2-(4-(2-(^18^F)fluoroethoxy)phenyl)-5,7-dimethylpyrazolo[1,5-a]pyrimidin-3-yl)acetamide | [75–81] | [82,83] |  | [84–90] |  |  |  |
| [^11^C]-DPA-713 | N,N-diethyl-2-(2-(4-methoxyphenyl)-5,7-dimethylpyrazolo[1,5-a]pyrimidin-3-yl)-acetamide | [5,91–94] | [95] | [59,96,97] |  |  | [98] |  |
| [^18^F]-FEPPA | [^18^F]-N-(2-(2-fluoroethoxy)benzyl)-N-(4-phenoxypyridin-3-yl) acetamide | [99–101] | [102–105] | [106–108] |  |  | [109] | [110–117] |
| [^18^F]-FEDAA1106 | *N*-(5-fluoro-2-phenoxyphenyl)-*N*-(2-[^18^F]fluoroethyl-5-methoxybenzyl)acetamide | [118] |  |  | [119] |  |  |  |
| [^18^F]-PBR111 | 2-(6-chloro-2-(4-(3-fluoropropoxy)phenyl)imidazo[1,2-a]pyridin-3-yl)-N,N-diethylacetamide |  |  |  | [64,120] |  |  | [121] |
| [^18^F]-PBR06 | [F-18]PBR06 [N-(2,5-dimethoxybenzyl)-2-(18)F-fluoro-N-(2-phenoxyphenyl)acetamide] |  |  |  | [122–124] |  |  |  |
| **Third Generation Tracers** | | | | | | | | |
| [^18^F]-GE-180 | (S)-N,N-diethyl-9-(2-[^18^F]fluoroethyl)-5-methoxy-2,3,4,9-tetrahydro-1H-carbazole-4-carboxamide | [125–127] |  |  | [128] |  |  |  |
| [^11^C]-ER176 | ^11^C-(*R*)-*N*-*sec*-butyl-4-(2-chlorophenyl)-*N*-methylquinazoline-2-carboxamide | [129] |  |  |  | [130] |  | [131] |

**Table S2. TSPO-PET and DCE-MRI Outcomes**

Reviewed studies have been organized based on disease and quantitative model used to derive reported outcomes. Symbols indicate the input function method used. 1T2k = reversible one-tissue compartment model; 2Tk4 = reversible two-tissue compartment model; 1TCM = one-tissue compartment model; 2TCM = two-tissue compartment model; MA1 = multilinear analysis; 2TCM-1K = two-tissue compartment model with a vascular component; SRTM = simplified reference tissue model; 2T3k = irreversible two-tissue compartment model; 3TCM = three-tissue compartment model.

| **Disease Condition** | | | | | | | |
| --- | --- | --- | --- | --- | --- | --- | --- |
| **Outcome** | **AD** | **PD** | **Epilepsy** | **MS** | **HD** | **Scz** | **Depression** |
| Total Distribution Volume (V_T_) | ***1T2k/2Tk4*** [79] ^*^ ***1TCM***  [91,92,94]**^*^ *2TCM***  [44–46,53,74,99–101,118]***^*^***  ***Logan Graphical Analysis***  [47,48,54,74,118,129]*^*^* | ***2TCM***  [56,102–105]^*^ ***Logan Graphical Analysis***  [58,95]^*^  ***WAP Multilinear Logan Graphical Analysis***  [55,57]^*^ | ***2TCM***  [59,60]**^*^** | ***12TK***  [84]^*^ ***2TCM***  [63,119,120]^*^  ***Logan Graphical Analysis***  [21]^∑^ [31,120]^*^ **MA1**  [61]^*^ |  | ***2TCM***  [37,109]^*^ ***2TCM-1K***  [69,70]^*^ ***Logan Graphical Analysis***  [98]^*^ | ***1TCM***  [73]^*^ ***2TCM***  [73,110–117,121]**^*^** ***MA1***  [73]**^*^** ***Logan Graphical Analysis***  [72]^*^  ***Graphical Analysis***  [131]**^*^** |
| Distribution volume ratio (DVR) | ***2TCM***  [44,46]^┼^  ***Logan Graphical Analysis***  [47] ***Logan Reference Tissue Model***  [13,14]^◇^ |  | ***Logan Reference Tissue Model***  [106,107]^◇^ | ***12TK***  [84]^*^ ***Logan Reference Tissue Model***  [22–24,26–30,32–34,85–89]^◇^ [62,64,66]^⌘^ ***SRTM***  [25]^◇^ | ***Logan Reference Kinetic Plot***  [68]^⌘^ | ***2TCM***  [37]^*^ ***2TCM-1K***  [69,70]^┼⌘^ |  |
| Binding Potential (BP_ND_) | ***Simplified Reference Region Model***  [18]^◇^  ***1TCM***  [91,92,94]^*⌘^ ***2TCM***  [81,118]**^*^ *1T2k/2T3k/2Tk4***  [79]**^*^** ***SRTM***  [1–10,16,17,52,93]^◇^  [79]^┼^ ***Logan Reference Tissue Model***  [81]^◇^ ***Multilinear reference tissue model***  [12,15]^◇^ | ***SRTM***  [83]^┼^  [19]^∑^  [20] ^◇^  ***Logan Reference Tissue Model***  [82]*^◇^* |  | ***12TK***  [84]^*^  **2TCM**  [119]^*^  **SRTM**  [90]^◇^ | ***SRTM***  [35]^◇^ | ***SRTM***  [38,71]^┼^ [39]^┼◇^  ***2TCM***  [36]^*^ | ***SRTM***  [40–42]^◇^ ***SRTM2***  [43]^◇^ |
| Standard uptake value ratio (SUVR) | [11,15,44,46,47,49–51,53,75,76,78,99,125,127]^┼^  [80]^┼⌘^ |  | [96,97,108]^┼^ | [63,65] ^◇^  [123,124,128]^⌘^ ***Glial activity load on PET (GALP)***  [122] | [67]^⌘^  [130]^┼^ |  |  |
| Rate constants (K1, k2, k3 , k4, an kb) | ***2TCM***  [118]^*^ |  |  | ***12TK***  [84]^*^ ***2TCM-1K*** [65]^*^ |  | ***2TCM***  [37]^*^ |  |
| Volume transfer constant (K_trans_) | ***Patlak Model***  [132,133]^†^ [134–136]^‡^  [137–141]^#^  [142] | ***Patlak Model***  [143]^#^ | ***2TCM***  [144]^**^ ***2TCM based on Extended Tofts Model***  [145]^#^  ***3TCM***  [146] | ***Modified Tofts model***  [147]^§^ ***Patlak Model*** [148,149]^#^ |  | ***Patlak Model***  [150]^#^ |  |
| Leakage Rate (K_i_) | ***Patlak Model***  [151–154]**^#^** |  |  | ***Patlak Model***  [155,156]^‡^ [149,157]^#^ |  |  |  |
| Rate transfer constant (K_EP_) |  |  | ***2TCM***  [144]^**^  ***3TCM***  [146] | ***Modified Tofts model***  [147]^§^ |  |  |  |
| Leakage Volume (V_L_) | ***Patlak Model***  [152]**^#^** |  |  |  |  |  |  |
| Fractional Plasma Volume (v_p_) | ***Patlak Model***  [138,151,153]^#^ [142] |  |  | ***Patlak Model***  [148]^#^ |  | ***Patlak Model***  [150]^#^ |  |
| Fractional interstitial volume (v_e_) |  |  | ***2TCM***  [144]^**^  ***3TCM***  [146] |  |  |  |  |
| Permeability Surface Product (PS) |  |  |  | ***1TCM***  [158]^§^ |  |  |  |
| EES fractional volume (Vb) |  |  |  | ***Modified Tofts model***  [147]^§^ |  |  |  |
| Total Permeability | ***Patlak Model***  [159] |  | ***Linear Dynamic Method*** |  |  |  |  |
| \| * arterial blood \| \| --- \| \| *◇* SVCA/Clustering Analysis \| \| ┼ cerebellum reference region \| \| ⌘ other reference region \| \| ∑ IDIF or Population-based AIF \| \| † AIF from common carotid artery \| \| ‡ AIF from internal carotid artery \| \| # VIF from superior sagittal sinus \| \| § AIF in middle cerebral artery \| \| ** Voxel-wise derived AIF \| | | | | | | | |

**Table S3. Literature search strings**

| **Disease Condition** | **PubMed Search String** |
| --- | --- |
| Alzheimer’s Disease | **TSPO-PET:** ((("positron-emission tomography"[MeSH Terms] OR ("positron-emission"[All Fields] AND "tomography"[All Fields]) OR "positron-emission tomography"[All Fields] OR ("positron"[All Fields] AND "emission"[All Fields] AND "tomography"[All Fields]) OR "positron-emission tomography"[All Fields] OR "PET Scan"[All Fields] OR "PET Imaging"[All Fields] OR "PET Scans"[All Fields] OR "PET Imagings"[All Fields]) AND (alzheimer's disease[All Fields] OR alzheimer*[All Fields]) AND ("translocator protein"[All Fields] OR "TSPO"[All Fields]) AND (2013/1/1:2025/1/1[pdat] AND "english"[Language])) NOT "animals"[MeSH Terms:noexp]) NOT (review[Filter] OR systematicreview[Filter] OR "Review"[Publication Type] OR "Systematic Review"[Publication Type])  **DCE-MRI:** (((dynamic contrast-enhanced[All Fields] OR DCE[All Fields] OR dynamic enhanced[All Fields]) AND (MRI[All Fields] OR magnetic resonance imaging[All Fields]) AND (alzheimer's disease[All Fields] OR alzheimer*[All Fields]) AND (2013/1/1:2025/1/1[pdat] AND "english"[Language])) NOT "animals"[MeSH Terms:noexp]) NOT (review[Filter] OR systematicreview[Filter] OR "Review"[Publication Type] OR "Systematic Review"[Publication Type]) |
| Parkinson’s Disease | **TSPO-PET:** ((("positron-emission tomography"[MeSH Terms] OR ("positron-emission"[All Fields] AND "tomography"[All Fields]) OR "positron-emission tomography"[All Fields] OR ("positron"[All Fields] AND "emission"[All Fields] AND "tomography"[All Fields]) OR "positron-emission tomography"[All Fields] OR "PET Scan"[All Fields] OR "PET Imaging"[All Fields] OR "PET Scans"[All Fields] OR "PET Imagings"[All Fields]) AND (parkinson's disease[All Fields]) AND ("translocator protein"[All Fields] OR "TSPO"[All Fields]) AND (2013/1/1:2025/1/1[pdat] AND "english"[Language])) NOT "animals"[MeSH Terms:noexp]) NOT (review[Filter] OR systematicreview[Filter] OR "Review"[Publication Type] OR "Systematic Review"[Publication Type])  **DCE-MRI:** (((dynamic contrast-enhanced[All Fields] OR DCE[All Fields] OR dynamic enhanced[All Fields]) AND (MRI[All Fields] OR magnetic resonance imaging[All Fields]) AND (parkinson's disease[All Fields]) AND (2013/1/1:2025/1/1[pdat] AND "english"[Language])) NOT "animals"[MeSH Terms:noexp]) NOT (review[Filter] OR systematicreview[Filter] OR "Review"[Publication Type] OR "Systematic Review"[Publication Type]) |
| Epilepsy | **TSPO-PET:** ((("positron-emission tomography"[MeSH Terms] OR ("positron-emission"[All Fields] AND "tomography"[All Fields]) OR "positron-emission tomography"[All Fields]  OR ("positron"[All Fields] AND "emission"[All Fields] AND "tomography"[All Fields])  OR "positron-emission tomography"[All Fields] OR "PET Scan"[All Fields] OR "PET Imaging"[All Fields] OR "PET Scans"[All Fields] OR "PET Imagings"[All Fields]) AND (epilep*[All Fields] OR seizure[All Fields]) AND ("translocator protein"[All Fields] OR "TSPO"[All Fields]) AND (2013/1/1:2025/1/1[pdat] AND "english"[Language])) NOT "animals"[MeSH Terms:noexp]) NOT (review[Filter] OR systematicreview[Filter] OR "Review"[Publication Type] OR "Systematic Review"[Publication Type])  **DCE-MRI:** (((dynamic contrast-enhanced[All Fields] OR DCE[All Fields] OR dynamic enhanced[All Fields]) AND (MRI[All Fields] OR magnetic resonance imaging[All Fields]) AND (epilep*[All Fields] OR seizure[All Fields] OR status epilepticus[All Fields) AND (2013/1/1:2025/1/1[pdat] AND "english"[Language])) NOT "animals"[MeSH Terms:noexp]) NOT (review[Filter] OR systematicreview[Filter] OR "Review"[Publication Type] OR "Systematic Review"[Publication Type]) |
| Multiple Sclerosis | **TSPO-PET:** ((("positron-emission tomography"[MeSH Terms] OR ("positron-emission"[All Fields] AND "tomography"[All Fields]) OR "positron-emission tomography"[All Fields] OR ("positron"[All Fields] AND "emission"[All Fields] AND "tomography"[All Fields]) OR "positron-emission tomography"[All Fields] OR "PET Scan"[All Fields] OR "PET Imaging"[All Fields] OR "PET Scans"[All Fields] OR "PET Imagings"[All Fields]) AND ("multiple sclerosis"[MeSH Terms] OR "multiple sclerosis"[All Fields]) AND ("translocator protein"[All Fields] OR "TSPO"[All Fields]) AND (2013/1/1:2025/1/1[pdat] AND "english"[Language])) NOT "animals"[MeSH Terms:noexp]) NOT (review[Filter] OR systematicreview[Filter] OR "Review"[Publication Type] OR "Systematic Review"[Publication Type])  **DCE-MRI:** (((dynamic contrast-enhanced[All Fields] OR DCE[All Fields] OR dynamic enhanced[All Fields]) AND (MRI[All Fields] OR magnetic resonance imaging[All Fields]) AND ("multiple sclerosis"[MeSH Terms] OR "multiple sclerosis"[All Fields]) AND (2013/1/1:2025/1/1[pdat] AND "english"[Language])) NOT "animals"[MeSH Terms:noexp]) NOT (review[Filter] OR systematicreview[Filter] OR "Review"[Publication Type] OR "Systematic Review"[Publication Type]) |
| Huntington’s Disease | **TSPO-PET:** ((("positron-emission tomography"[MeSH Terms] OR ("positron-emission"[All Fields] AND "tomography"[All Fields]) OR "positron-emission tomography"[All Fields] OR ("positron"[All Fields] AND "emission"[All Fields] AND "tomography"[All Fields]) OR "positron-emission tomography"[All Fields] OR "PET Scan"[All Fields] OR "PET Imaging"[All Fields] OR "PET Scans"[All Fields] OR "PET Imagings"[All Fields]) AND (huntington's disease[All Fields]) AND ("translocator protein"[All Fields] OR "TSPO"[All Fields]) AND (2013/1/1:2025/1/1[pdat] AND "english"[Language])) NOT "animals"[MeSH Terms:noexp]) NOT (review[Filter] OR systematicreview[Filter] OR "Review"[Publication Type] OR "Systematic Review"[Publication Type])  **DCE-MRI:** (((dynamic contrast-enhanced[All Fields] OR DCE[All Fields] OR dynamic enhanced[All Fields]) AND (MRI[All Fields] OR magnetic resonance imaging[All Fields]) AND (huntington's disease[All Fields]) AND (2013/1/1:2025/1/1[pdat] AND "english"[Language])) NOT "animals"[MeSH Terms:noexp]) NOT (review[Filter] OR systematicreview[Filter] OR "Review"[Publication Type] OR "Systematic Review"[Publication Type]) |
| Schizophrenia | **TSPO-PET:** ((("positron-emission tomography"[MeSH Terms] OR ("positron-emission"[All Fields] AND "tomography"[All Fields]) OR "positron-emission tomography"[All Fields] OR ("positron"[All Fields] AND "emission"[All Fields] AND "tomography"[All Fields]) OR "positron-emission tomography"[All Fields] OR "PET Scan"[All Fields] OR "PET Imaging"[All Fields] OR "PET Scans"[All Fields] OR "PET Imagings"[All Fields]) AND (schizophrenia[All Fields] OR psychosis[All Fields]) AND ("translocator protein"[All Fields] OR "TSPO"[All Fields]) AND (2013/1/1:2025/1/1[pdat] AND "english"[Language])) NOT "animals"[MeSH Terms:noexp]) NOT (review[Filter] OR systematicreview[Filter] OR "Review"[Publication Type] OR "Systematic Review"[Publication Type])  **DCE-MRI:** (((dynamic contrast-enhanced[All Fields] OR DCE[All Fields] OR dynamic enhanced[All Fields]) AND (MRI[All Fields] OR magnetic resonance imaging[All Fields]) AND (schizophrenia[All Fields] OR psychosis[All Fields]) AND (2013/1/1:2025/1/1[pdat] AND "english"[Language])) NOT "animals"[MeSH Terms:noexp]) NOT (review[Filter] OR systematicreview[Filter] OR "Review"[Publication Type] OR "Systematic Review"[Publication Type]) |
| Depression | **TSPO-PET:** ((("positron-emission tomography"[MeSH Terms] OR ("positron-emission"[All Fields] AND "tomography"[All Fields]) OR "positron-emission tomography"[All Fields] OR ("positron"[All Fields] AND "emission"[All Fields] AND "tomography"[All Fields]) OR "positron-emission tomography"[All Fields] OR "PET Scan"[All Fields] OR "PET Imaging"[All Fields] OR "PET Scans"[All Fields] OR "PET Imagings"[All Fields]) AND (depression[All Fields] OR depress*[ All Fields] OR major depressive disorder[All Fields]) AND ("translocator protein"[All Fields] OR "TSPO"[All Fields]) AND (2013/1/1:2025/1/1[pdat] AND "english"[Language])) NOT "animals"[MeSH Terms:noexp]) NOT (review[Filter] OR systematicreview[Filter] OR "Review"[Publication Type] OR "Systematic Review"[Publication Type])  **DCE-MRI:** (((dynamic contrast-enhanced[All Fields] OR DCE[All Fields] OR dynamic enhanced[All Fields]) AND (MRI[All Fields] OR magnetic resonance imaging[All Fields]) AND (depression[All Fields] OR depress*[ All Fields] OR major depressive disorder[All Fields]) AND (2013/1/1:2025/1/1[pdat] AND "english"[Language])) NOT "animals"[MeSH Terms:noexp]) NOT (review[Filter] OR systematicreview[Filter] OR "Review"[Publication Type] OR "Systematic Review"[Publication Type]) |

**Table S4. Association between TSPO outcomes and AD disease pathology**

Study findings are summarized based on CSF or PET-derived Aβ, CSF or PET-derived tau, neurodegeneration, and cognitive outcomes. Summarized findings are reported for the combined group (noted under the “study group” column), unless otherwise specified. N sizes are reported for separate groups, when possible, otherwise the total group N size is reported.

AD = Alzheimer’s disease; Aβ = amyloid; SUVR = standard uptake value ratio; BP = binding potential; MCI = mild cognitive impairment; BP_ND_ = non-displaceable binding potential; HCs = healthy controls; DVR = distribution volume ratio; APOE = Apolipoprotein E4; ROI = region of interest; BMI = body mass index; V_T_ = distribution volume; hs-CRP = high-sensitivity C-reactive protein; TREM2 = Triggering Receptor Expressed on Myeloid cells 2; aMCI = amnestic mild cognitive impairment; HIPP = hippocampus; MCI-EOAD = mild cognitive impairment – early onset Alzheimer’s disease; CSF = cerebrospinal fluid; pTau181 = phosphorylated tau; tTau = total tau protein; ACC = anterior cingulate cortex; HAB = high affinity binder; MAB = mixed affinity binder; NfL = neurofilament light chain; CDR = clinical dementia rating; MMSE = Mini Mental State Examination; RAVLT = Rey Auditory Verbal Learning Test; ADRC-PACC = Alzheimer Disease Research Center- Preclinical Alzheimer Cognitive Composite ; APCC = preclinical cognitive composite; MoCA = Montreal Cognitive Assessment; V_T_/*f*_p_ = total distribution volume corrected by the plasma free fraction; SRT-DR = Selective Reminding Test-Delayed Recall; ADAS J-cog = Alzheimer's Disease Assessment Scale- Cognitive subscale (Japanese version);

|  | **Study Group** | **Tracer** | **Finding** | **Citation** |
| --- | --- | --- | --- | --- |
| **Aβ Outcomes** | | | | |
| **PET** | Aβ+ AD (N=8) | [^11^C]-PK11195 | Voxel-wise ↑ [^11^C]-PiB Aβ-PET SUVR associated with ↑ [^11^C]-PK11195 BP at baseline and less extensively at follow-up | [3,160] |
|  | MCI (N=10) & AD (N=10) | [^11^C]-PK11195 | Voxel-wise ↑ [^11^C]-PiB Aβ-PET SUVR associated with ↑ [^11^C]-PK11195 BP. The number of voxels with positive correlations was lower for MCI subjects relative to AD | [2] |
|  | Aβ+/- MCI (N=8) & Aβ+ AD (N=8) | [^11^C]-PK11195 | Voxel-wise ↑ [^11^C]-PiB Aβ-PET SUVR associated with ↑ [^11^C]-PK11195 BP at baseline and follow up. | [4] |
|  | Aβ+ MCI (N=26) | [^11^C]-PK11195 | Voxel-wise associations between ↑ [^11^C]-PiB Aβ-PET SUVR and ↑ [^11^C]-PK11195 BP_ND_ | [6] |
|  | high Aβ MCI/AD (N=16) | [^11^C]-PK11195 | Voxel-wise associations between ↑ [^11^C]-PiB Aβ-PET SUVR and ↑ [^11^C]-PK11195 BP_ND_ | [7] |
|  | low Aβ MCI (N=15) & high Aβ MCI (N=27) | [^11^C]-PK11195 | ↑ [^11^C]-PiB Aβ-PET SUVR associated with ↑ [^11^C]-PK11195 BP_ND_ at baseline and at a 2-year follow-up for low Aβ MCI subjects who had increased Aβ load over two years (N=7), but not for those with stable Aβ load (N=8).  ↑ [^11^C]-PiB Aβ-PET SUVR associated with ↑ [^11^C]-PK11195 BP_ND_ at baseline and at a 2-year follow-up (N=23) for high Aβ MCI | [10] |
|  | Aβ+ AD (N=19) | [^11^C]-PK11195 | No association between [^11^C]-PiB Aβ-PET SUVR and [^11^C]-PK11195 BP_ND_ | [12] |
|  | HCs with variable Aβ load: APOE non-carriers (ε3/ε3) (N=20)  APOE heterozygotes (ε3/ε4) (N=21) APOE homozygotes (ε4/ε4) (N=19) | [^11^C]-PK11195 | ↑ [^11^C]-PiB Aβ-PET SUVR associated with ↑ [^11^C]-PK11195 DVR in APOE (ε4/ε4) but not in APOE (ε3/ε4) in ROI analysis. ↑ [11C]-PiB Aβ-PET binding associated with ↑ [^11^C]-PK11195 binding in APOE (ε4/ε4) and APOE (ε3/ε4) in voxel-wise analysis ↑ [^11^C]-PiB Aβ-PET SUVR associated with ↓ [^11^C]-PK11195 DVR in APOE (ε3/ε3) in ROI analysis | [14] |
|  | Aβ+ (N=34) & Aβ- old HCs (N=26) | [^11^C]-PK11195 | ↑ Insulin resistance & BMI associated with [^11^C]-PK11195 DVR in the total study population ↑ Insulin resistance associated with ↑ [^11^C]-PK11195 DVR in Aβ+/- APOE non-carriers (N=20) and homozygotes (N=19) | [13] |
|  | MCI (N=10) & AD (N=19) | [^11^C]-PBR28 | ↑ [^11^C]-PiB Aβ-PET V_T_ associated with ↑ [^11^C]-PBR28 V_T_ | [45] |
|  | Aβ+ MCI/AD (N=14) & HCs (N=8) | [^11^C]-PBR28 | No correlation between % change in [^11^C]-PiB Aβ-PET SUVR and % change in [^11^C]-PBR28 SUVR | [46] |
|  | Aβ+ Posterior Cortical Atrophy (N=11) & AD (N=11) | [^11^C]-PBR28 | ↑ [^11^C]-PiB Aβ-PET SUVR associated with ↑ [^11^C]-PBR28 SUVR in the precuneus | [53] |
|  | Aβ+/- MCI (N=16) & AD (N=16) | [^11^C]-PBR28 | Voxel-wise ↑ [18F]-flutemetamol Aβ-PET V_T_ associated with ↑[^11^C]-PBR28 V_T_;  The same relationship was reported in solely Aβ+ MCI (N=9) and Aβ+ AD (N=14), respectively | [48] |
|  | Aβ+/- AD (N=23/10) & Aβ+/- HCs (N=7/17) | [^11^C]-PBR28 | Aβ positivity associated with ↑ [^11^C]-PBR28 SUVR | [50] |
|  | MCI (N=28), AD (N=16), & young HCs (N = 22) & old HCs (N=64) | [^11^C]-PBR28 | ↑ [^18^F]-Flutafuranol Aβ-PET associated with ↑ [^11^C]-PBR28 SUVR | [51] |
|  | Aβ+ (N=25) & Aβ- old HCs (N=29) | [^11^C]-PBR28 | ↑ [^11^C]-PiB Aβ-PET SUVR associated with ↑ [^11^C]-PBR28 SUVR in Aβ-, but not Aβ+ participants ↑ Insulin resistance, BMI and hs-CRP associated with ↑ [^11^C]-PBR28 SUVR in all participants | [47] |
|  | MCI/mild AD (N=22) & HCs (N=19) | [^11^C]-PBR28 | ↑ [18F]-FBB Aβ-PET SUVR associated with ↑ [^11^C]-PBR28 SUVR | [49] |
|  | Aβ+ Prodromal (N=34) & Dementia AD (N=24) | [^18^F]-DPA-714 | ↑ [^11^C]-PiB Aβ-PET SUVR associated with ↑ [^18^F]-DPA-714 SUVR | [77] |
|  | Aβ+ Prodromal (N=21) & Dementia AD (N=15) | [^18^F]-DPA-714 | No association between baseline [^11^C]-PiB Aβ-PET SUVR and % change in [^18^F]-DPA-714 SUVR | [78] |
|  | MCI (N = 8) & TREM2 p.R47H carriers (N=8) | [^18^F]-DPA-714 | Aβ positivity was not significantly associated with [^18^F]-DPA-714 DVR across Braak stage regions | [81] |
|  | aMCI (N=28), AD (N=29), & HCs (N=28) | [^18^F]-DPA-714 | ↑ [^18^F]-Florbetapir Aβ-PET SUVR associated with ↑ [^18^F]-DPA-714 SUVR  blood CD200 expression levels mediated the association between ↑ [^18^F]-Florbetapir Aβ-PET SUVR and ↑ [^18^F]- DPA-714 SUVR | [76] |
|  | aMCI (N=11) | [^18^F]-FEPPA | ↑ [^11^C]-PIB Aβ-PET DVR associated with ↑ HIPP [^18^F]-FEPPA V_T_ | [99] |
|  | Prodromal AD & AD females (N=29) | [^18^F]-GE-180 | ↑BMI associated with ↑Aβ-independent [^18^F]-GE-180 PET z-score. The same association was not observed in male AD subjects (N=20) | [125] |
|  | Aβ+/- AD (N = 18) | [^18^F]-GE-180 | ↑ [^18^F]-flutemetamol Aβ-PET z-scores associated with ↑[^18^F]-GE-180 PET z-scores | [127] |
|  | MCI-EOAD (N=23) & HCs (N = 23) | [^11^C]-ER176 | ↑ [^18^F]-FBB/[^11^C]-PiB Aβ-PET SUVR associated with ↑ [^11^C]-ER176 SUVR in all binding affinity groups for all participants ↑ [^18^F]-FBB/[^11^C]- PiB Aβ-PET SUVR associated with ↑ [^11^C]-ER176 SUVR in 11/22 MCI-EOAD | [129] |
| **CSF**  **Aβ_42_**  **Aβ_40_**  **Aβ_42_/Aβ_40_ ratio** | AD & HCs (N=16) | [^11^C]-PK11195 | ↓ CSF Aβ_42_ and Aβ_42_/ Aβ_40_ ratio associated with ↑ [^11^C]-PK11195 SUVR | [11] |
|  | HCs with variable Aβ load: APOE non-carriers (ε3/ε3) (N=20)  APOE heterozygotes (ε3/ε4) (N=21) APOE homozygotes (ε4/ε4) (N=19) | [^11^C]-PK11195 | No association between plasma Aβ_1-42_/ Aβ_1-40_ ratio and global [^11^C]-PK11195 DVR | [14] |
|  | Aβ/p-tau 181+ MCI (N=5) & AD (N=14) | [^11^C]-DPA-713 | No association between CSF Aβ_42_/_40_ ratio and [^11^C]-DPA713 BP_ND_ | [92] |
|  | aMCI (N=11) & AD (N=10) HABs | [^18^F]-FEPPA | ↓ CSF Aβ_42_ associated with ↑ [^18^F]-FEPPA V_T_ | [101] |
| **Tau Outcomes** | | | | |
| **PET** | high Aβ MCI/AD (N=16) | [^11^C]-PK11195 | No ROI or voxel-wise association between [^18^F]-flortaucipir tau-PET SUVR and [^11^C]-PK11195 BPND | [7] |
|  | high Aβ MCI (N=22) | [^11^C]-PK11195 | The association between [^11^C]-PiB Aβ-PET SUVR and [^11^C]-PK11195 BP_ND_ disappeared when controlling for [18F]-flortaucipir tau-PET SUVR  ↑ [^18^F]-flortaucipir tau-PET SUVR associated with ↑ [^11^C]-PK11195 BP_ND_ at baseline and at a 2-year follow up before and after including [^11^C]-PiB Aβ-PET SUVR as a covariate | [10] |
|  | Aβ+ MCI (N=14), Aβ- MCI (N=10), AD (N=15), & HCs (N=27) | [^11^C]-PK11195 | ↑ [^18^F]-flortaucipir tau-PET BP_ND_ associated with ↑ [^11^C]-PK11195 BP_ND_ | [52] |
|  | Aβ+/- MCI (N=16) & Aβ+/- AD (N=16) | [^11^C]-PBR28 | Voxel-wise association between ↑ [18F]-flortaucipir tau-PET V_T_ and ↑ [^11^C]-PBR28 V_T_. The same relationship was reported in solely Aβ+ MCI (N=9) and Aβ+ AD (N=14), respectively. | [48] |
|  | Aβ+/- AD (N=9/14) & Aβ+/- HCs (N=7/12) | [^11^C]-PBR28 | ↑ [^18^F]-MK-6240 tau-PET SUVR associated with ↑ [^11^C]-PBR28 SUVR | [50] |
|  | Cognitively impaired old (N=22) & old HCs (N=34) | [^11^C]-PBR28 | ↑ [^18^F]-MK-6240 tau-PET SUVR associated with ↑ [^11^C]-PBR28 SUVR ↑ % change in Braak II-VI [^18^F]-MK-6240 tau-PET SUVR associated with ↑ baseline Braak I [^11^C]-PBR28 SUVR | [51] |
|  | MCI/mild AD (N=22) & HCs (N=19) | [^11^C]-PBR28 | ↑ Braak III-VI [^18^F]-MK-6240 tau-PET SUVR associated with ↑ [^11^C]-PBR28 SUVR ↑ [^11^C]-PBR28 SUVR mediated the association between ↑ [^18^F]-MK-6240 tau-PET SUVR in Braak I-II and Braak III-IV areas. | [49] |
|  | MCI (N = 8) & TREM2 p.R47H carriers (N=8) | [^18^F]-DPA-714 | Braak I [^18^F]-flortaucipir tau-PET SUVR was not significantly associated with [^18^F]-DPA-714 DVR across Braak stage regions | [81] |
|  | Aβ+ AD (N=20) | [^11^C]-DPA-713 | ↑ [^11^C]-PBB3 tau-PET BP_ND_ associated with ↑ [^11^C]-DPA-713 BP_ND_ in the parahippocampus | [93] |
|  | Tau+/- AD (N = 18) | [^18^F]-GE-180 | ↑ [^18^F]-PI-2620 tau-PET z-scores associated with ↑[^18^F]-GE-180 PET z-scores | [127] |
|  | Prodromal AD & AD females (N=15) | [^18^F]-GE-180 | ↑ Braak II area [^18^F]-PI-2620 tau-PET z-score associated with ↑Aβ -independent [^18^F]-GE-180 PET z-score. The same association was not observed in male AD subjects (N=10) | [125] |
|  | MCI-EOAD (N=21) & HCs (N = 19) | [^11^C]-ER176 | ↑ [^18^F]-flortaucipir tau-PET SUVR associated with ↑ [^11^C]-ER176 SUVR in all binding affinity groups for all participants ↑ [^18^F]-flortaucipir tau-PET SUVR associated with ↑ [^11^C]-ER176 SUVR in 16/21 MCI-EOAD | [129] |
| **CSF**  **tTau**  **pTau** | AD & HCs (N=16) | [^11^C]-PK11195 | No association between CSF pTau181 or tTau and [^11^C]-PK11195 SUVR | [11] |
|  | Aβ+ AD (N=17) | [^11^C]-PK11195 | No association between CSF tau and [^11^C]-PK11195 BP_ND_ in the ACC | [12] |
|  | Aβ/p-tau 181+ MCI (N=5) & AD (N=14) | [^11^C]-DPA-713 | No association between CSF pTau181 and [^11^C]-DPA713 BP_ND_ | [92] |
| **Neurodegeneration Outcomes** | | | | |
| **Cortical Atrophy** | HCs with all three APOE genotypes & variable Aβ load:  APOE non-carriers (ε3/ε3) (N=20)  APOE heterozygotes (ε3/ε4) (N=21) APOE homozygotes (ε4/ε4) (N=19)" | [^11^C]-PK11195 | ↓ HIPP and entorhinal volume associated with ↑ [^11^C]-PK11195 DVR | [14] |
|  | AD (N=8) | [^11^C]-PK11195 | Voxel-wise association between ↑ HIPP volume and ↓ [^11^C]-PK11195 BP_ND_. | [17] |
|  | MCI (N=10) & AD (N=19) | [^11^C]-PBR28 | ↓ Grey matter volumes associated with ↑ [^11^C]-PBR28 V_T_ | [45] |
|  | Aβ+ MCI & AD (N=14) | [^11^C]-PBR28 | ↑ % reduction in gray matter volume associated with ↑ % change [^11^C]-PBR28 SUVR | [46] |
|  | Aβ+/- MCI (N=37) | [^11^C]-PBR28 | Voxel-wise association between ↑ grey matter volume and ↑ [11C]-PBR28 V_T_. ↑ HIPP volume associated with ↑ [^11^C]-PBR28 V_T_. | [54] |
|  | MCI/mild AD (N=22) & HCs (N=19) | [^11^C]-PBR28 | ↑ [^11^C]-PBR28 SUVR mediated the association between ↑ [^18^F]- MK-6240 tau-PET SUVR and neurodegeneration in Braak III-IV areas | [49] |
|  | Aβ+ prodromal (N=38) & dementia AD (N=26) | [^18^F]-DPA-714 | ↑ Grey matter volume associated with ↑ global [^18^F]-DPA-714 SUVR in HAB + MAB (prodromal N=34 and dementia AD N=24) and prodromal AD groups (with all binding affinities). | [77] |
|  | Aβ+ prodromal (N=33) & dementia AD (N=19) | [^18^F]-DPA-714 | ↑ HIPP and cortical gray matter volumes associated with ↑ [^18^F]-DPA-714 SUVR at baseline ↓ % HIPP volume decr., but not cortical grey matter, associated with ↑ [^18^F]-DPA-714 SUVR at baseline  ↑ % left HIPP volume decr. associated with ↑ % change in [^18^F]-DPA-714 SUVR in the longitudinal whole AD patient group (N=21) and prodromal AD group (N=15) | [78] |
|  | MCI-EOAD (N=25) & HCs (N = 23) | [11C]-ER176 | ↓ Cortical volume associated with ↑ [^11^C]-ER176 SUVR in MAB and HAB in the whole cohort ↓ Cortical volume associated with ↑ [^11^C]-ER176 SUVR in 7/25 MCI-EOAD | [129] |
| **Axonal Degeneration** | Aβ+ MCI/AD (N=27) | [^11^C]-PK11195 | ↓ CSF NfL associated with ↑ [^11^C]-PK11195 BP_ND_ | [9] |
| **Cortical Thickness** | Aβ+ MCI/AD (N=27) | [^11^C]-PK11195 | No association between cortical thickness and [^11^C]-PK11195 BP_ND_ | [9] |
|  | Aβ+ Posterior Cortical Atrophy (N=11) & AD (N=11) | [^11^C]-PBR28 | ↓ Cortical thickness associated with ↑ [^11^C]-PBR28 SUVR | [53] |
| **[^18^F]-FDG Metabolism** | Aβ+ AD (N=8) | [^11^C]-PK11195 | Voxel-wise association between ↓ [^18^F]-FDG metabolism and ↑ [^11^C]-PK11195 BP | [3] |
|  | AD (N=8) | [^11^C]-PK11195 | Voxel-wise association between ↑ HIPP glucose metabolic rate and  ↓ [^11^C]-PK11195 BP_ND_. | [17] |
|  | MCI (N=10) & AD (N=10) | [^11^C]-PK11195 | Voxel-wise association between ↑ [^11^C]-PK11195 BP and ↓ [^18^F]-FDG metabolism | [2] |
|  | EOAD (N=12) | [^11^C]-PK11195 | ↑ [^11^C]-PK11195 BP associated with ↑ [^18^F]-FDG hypometabolism | [18] |
| **Cognitive Outcomes** | | | | |
| **Clinical disease severity (CDR) & Cognition** | prodromal AD (N=10), AD (N=19), & HCs (21) | [^11^C]-PK11195 | No association between cognition and [^11^C]-PK11195 BP_ND_ at baseline | [1] |
|  | AD (N=10) | [^11^C]-PK11195 | ↓ MMSE associated with voxel-wise ↑ [^11^C]-PK11195 BP | [2] |
|  | AD (N=10) & old HCs (N=10) | [^11^C]-PK11195 | No association between MMSE and [^11^C]-PK11195 BP_ND_ | [5] |
|  | probable AD & Aβ+ aMCI (n=16) | [^11^C]-PK11195 | ↑ Episodic memory associated with ↓ [^11^C]-PK11195 BP_ND_ | [8] |
|  | high Aβ MCI/AD (N=16) | [^11^C]-PK11195 | No association between MMSE and [^11^C]-PK11195 BP_ND_ | [7] |
|  | Aβ+ MCI/AD(N=27) | [^11^C]-PK11195 | No association between MMSE or CDR and [^11^C]-PK11195 BP_ND_ | [9] |
|  | Aβ+ MCI (N=14), Aβ- MCI (N=10), AD (N=15), & HCs (N=27) | [^11^C]-PK11195 | ↓ RAVLT score associated with ↑ [^11^C]-PK11195 BP_ND_. The effect of [^11^C]-PK11195 BP_ND_ on cognition was mediated by grey matter density. No effect modification was found for [^11^C]-PK11195 BP_ND_ on the association between [^18^F]-AV1451 tau-PET binding on cognition. | [52] |
|  | AD & HCs (N=16) | [^11^C]-PK11195 | ↑ [^11^C]-PK11195 SUVR predicted decline in Knight ADRC-PACC for the whole cohort and additionally in global composite scores for a sub-cohort (N=16) | [11] |
|  | HCs with variable Aβ load: APOE non-carriers (ε3/ε3) (N=20)  APOE heterozygotes (ε3/ε4) (N=21) APOE homozygotes (ε4/ε4) (N=19) | [^11^C]-PK11195 | No association between APCC scores and global [^11^C]-PK11195 DVR | [14] |
|  | MCI (N = 28) | [^11^C]-PK11195 | ↓ MoCA, ↓MMSE, and ↑ CDR associated with ↑ [^11^C]-PK11195 BP at baseline and 2 yr follow up  The association between [^11^C]-PiB Aβ-PET SUVR and MoCA as well as with CDR is mediated by [^11^C]-PK11195 BP at 2 yr follow up | [16] |
|  | AD (N=8) | [^11^C]-PK11195 | ↓ MMSE associated with ↑ [^11^C]-PK11195 BP_ND_ | [17] |
|  | MCI (N=10) & AD (N=19) | [^11^C]-PBR28 | ↓ MMSE and ↑ CDR associated with ↑ [^11^C]-PBR28 V_T_ | [45] |
|  | Aβ+ MCI (N=11), Aβ+ AD (N=25) & HCs (N=21) | [^11^C]-PBR28 | ↑ CDR associated with ↑ residual of [11C]-PBR28 V_T_/*f_p_*, SUVR, and DVR | [44] |
|  | Aβ+ MCI & AD (N=14) | [^11^C]-PBR28 | ↑ CDR associated with ↑ [^11^C]-PBR28 SUVR | [46] |
|  | Aβ+ AD patients (N=23) & Aβ+ HCs (N=7) | [^11^C]-PBR28 | ↓ MMSE associated with ↑ [^11^C]-PBR28 SUVR | [50] |
|  | MCI/mild AD (N=22) & HCs (N=19) | [^11^C]-PBR28 | ↓ MMSE, SRT-DR, executive function, and language scores associated with ↑ [^11^C]-PBR28 SUVR ↑ [^11^C]-PBR28 SUVR mediated the association between ↑ neurodegeneration in Braak (I-IV) areas and ↓ MMSE | [49] |
|  | AD (N=10) & HCs (N=7) | [^18^F]-FEMPA | No association between MMSE and [^18^F]-FEMPA V_T_ | [74] |
|  | Aβ+ prodromal (N=34) & dementia AD (N=24) | [^18^F]-DPA-714 | ↑MMSE associated with ↑ global [^18^F]-DPA-714 SUVR | [77] |
|  | Aβ+ prodromal (N=33) & dementia AD (N=19) | [^18^F]-DPA-714 | ↑ MMSE associated with ↑ [^18^F]-DPA-714 GCI at baseline  ↓ % MMSE decr. associated with ↑ [^18^F]-DPA-714 SUVR in the whole AD group at baseline, but not for the prodromal AD group ↓ % CDR decr., ↓ % long-term memory decr., ↓ % temporospatial orientation decr. and ↓ % short-term memory decr. associated with ↑ [^18^F]-DPA-714 SUVR in prodromal AD at baseline  ↑ % MMSE decr. and ↑ % CDR incr. associated with ↑ % change in [^18^F]-DPA-714 SUVR in the longitudinal whole AD patient group (N=21) and prodromal AD group (N=15) | [78] |
|  | MCI (N = 29) | [^18^F]-DPA-714 | No association between [^18^F]-DPA-714 SUVR and cognitive outcomes when correcting for multiple comparisons. Without correction, ↓ memory recall correlated with ↑ temporal [^18^F]-DPA-714 SUVR. Associations between [^18^F]-DPA-714 SUVR and cognitive outcomes were also reported at the voxel level. | [80] |
|  | aMCI (N=28), AD (N=29), & HCs (N=28) | [^18^F]-DPA-714 | ↓ MMSE and MoCA and ↑ CDR associated with ↑ global [^18^F]-DPA-714 SUVR  blood CD200 expression levels mediated the association between ↓ MMSE and ↑ [^18^F]-DPA-714 SUVR | [76] |
|  | AD (N=7) & elderly HCs(N=12) | [^11^C]-DPA-713 | ↓ MMSE associated with ↑ [^11^C]-DPA-713 BP_ND_ | [5] |
|  | Aβ+ AD (N=15) | [^11^C]-DPA-713 | Annual change in ADAS J-cog and memory scores significantly associated with [^11^C]-DPA-713 BP_ND_ | [94] |
|  | Aβ/p-tau 181+ MCI (N=5) & AD (N=14) | [^11^C]-DPA-713 | No association between MMSE or ADAS J-cog score and [^11^C]-DPA713 BP_ND_ | [92] |
|  | AD (N=21) | [^18^F]-FEPPA | No association between CDR and [^18^F]-FEPPA V_T_ ↓ visuospatial and language score associated with ↑ [^18^F]-FEPPA V_T_ | [100] |
|  | aMCI (N=11) | [^18^F]-FEPPA | No association between MMSE or MoCA and [^18^F]-FEPPA V_T_ | [99] |
|  | Aβ+ AD (N=32) | [^18^F]-GE-180 | Quadratic association between MMSE and CDR and anterior medial temporal lobe [^18^F]-GE-180 SUVR | [126] |
|  | MCI-EOAD (N=25) & HCs (N = 23) | [^11^C]-ER176 | ↓ MMSE & DemTect scores associated with ↑ [^11^C]-ER176 in the whole cohort | [129] |

**Table S5. Association between DCE-MRI outcomes and AD disease pathology**

Study findings are summarized based on cognitive, neurodegeneration, CSF or PET-derived Aβ, and CSF or PET-derived tau outcomes. Summarized findings are reported for the combined group (noted under the “study group” column), unless otherwise specified. N sizes are reported for separate groups, when possible, otherwise the total group N size is reported.

MCI = mild cognitive impairment; AD = Alzheimer’s disease; CSF = cerebrospinal fluid; tTau = total tau protein; K_trans_ = Volume transfer constant; pTau = phosphorylated tau; Aβ = amyloid; HCs = healthy controls; DVR = distribution volume ratio; SCCA = Sparse canonical correlation analysis ; SUVR = standard uptake value ratio; K_i_ = Leakage Rate; V_L_ = Leakage Volume; GM = Gray Matter; WM = white matter; NAWM = normal appearing white matter; WMH = white matter hyperintensity; SCI = subjective cognitive impairment; V_p_ = fractional plasma volume. HIPP = hippocampus; MMSE = Mini Mental State Examination; PHC = parahippocampus; CDR = clinical dementia rating; PHG = parahippocampal gyrus; APOE = Apolipoprotein E4; MoCA = Montreal Cognitive Assessment; vMCI = vascular mild cognitive impairment;

CGM = cortical grey matter; DGM = deep grey matter;

| **Study Group** | **Finding** | **Citation** |
| --- | --- | --- |
| **Amyloid & Tau PET and CSF outcomes** | | |
| Aβ+ MCI/AD (N=29) & Aβ- MCI/AD (N=33) | ↑ CSF tTau associated with ↑K_trans_ in the whole cohort ↓ CSF AB_40_ & CSF Ptau181 associated with ↑ K_trans_ in the Aβ+ group ↑ CSF AB_42_/AB_40_ ratio associated with ↑ K_trans_ in the Aβ+ group ↑ tTau associated with ↑ K_trans_ in the Aβ- group | [139] |
| Old HCs (N=31) | No significant association between global PiB index and averaged K_trans_ (across 20 ROIs associated with ↑ BBB dysfunction in old HCs) Sparse canonical correlation analysis (SCCA) showed a pattern of regional positive and negative associations between temporal and parietal [^11^C]-PIB Aβ-PET DVR and K_trans_ in the insula, temporal, and parietal cortices. SCCA also showed positive association between [^18^F]-Flortaucipir tau-PET SUVR and K_trans_ in lateral temporal and medial parietal regions. | [133] |
| **Neurodegeneration Outcomes** | | |
| MCI (N=34), AD (N=14), and HCs (N=32) | ↑ K_i_ and V_l_ associated with ↑ total GM, total WM, cortex, Deep GM, NAWM, and WMH volume | [152] |
| SCI, MCI, & AD (N=132) | ↓ K_trans_ and V_p_ associated with ↑ choroid plexus volume | [141] |
| Aβ+ MCI/AD (N=29) | ↓ HIPP volume associated with ↑ K_trans_ in the Aβ+ group | [139] |
| MCI (N=21), AD (N=26), and HCs (N=44) | No association between HIPP K_trans_ and WMH | [135] |
| Aβ+/- HCs (N=44) and MCI/AD (N=11) | ↑ Entorhinal free water associated with ↑ entorhinal K_trans_ No association between cortical thickness or volume and K_trans_ in any region | [137] |
| **Cognitive Outcomes** | | |
| MCI (N=9), AD (N=7), and HCs (N=17) | ↓MMSE associated with ↑ V_l_ in deep GM and cortex. | [153] |
| Aβ+/- patients CDR = 0 and CDR = 0.5 (N = 65) | ↑ Regional HIPP, PHC and HIPP subfields K_trans_ remained a significant predictor of CDR status and # of impaired cognitive domains (N=70) after controlling for CSF Aβ_1–42_and pTau | [134] |
| Patients with CDR = 0 (N=193) and  CDR = 0.5 (N = 32) | HIPP and PHG K_trans_ remained a significant predictor of CDR status in APOE4 homozygotes accounting for age, sex, education, CSF Aβ and pTau status, and HIPP and PHG volumes | [136] |
| MCI (N=34), AD (N=14), and HCs (N=32) | K_i_ was indirectly associated with information processing speed when accounting for WMH volume in a mediation analysis | [152] |
| HCs (N = 57) | ↑ decline in delayed recall associated with ↑ Ki in the WM and GM | [154] |
| vMCI (N=26) and HC (N = 21) | ↓MoCA associated with ↑ K_trans_ and V_p_ in NAWM, WMH, CGM, and DGM.  ↓MoCA associated only with ↑ K_trans_ in WMH and V_p_ in NAWM and CGM following adjustment for age, sex, vascular risk factors, and years of education. | [138] |
| Female only MCI (N = 24) | Occipital cortex K_trans_ associated with MMSE score | [140] |
| Aβ+ MCI/AD (N=29) & Aβ- MCI/AD (N=33) | No association between MMSE score and cortical K_trans_ | [139] |
| Aβ+/- CN (N=44) and MCI/AD (N=11) | No associations between cognitive scores and K_trans_ values in GM, WM, HIPP, entorhinal, or PHC | [137] |

**References**

1. Schuitemaker A, Kropholler MA, Boellaard R, et al. Microglial activation in Alzheimer’s disease: an (R)-[11C] PK11195 positron emission tomography study. Neurobiology of aging. 2013;34(1):128–36.

2. Fan Z, Aman Y, Ahmed I, et al. Influence of microglial activation on neuronal function in Alzheimer’s and Parkinson’s disease dementia. Alzheimer’s & Dementia. 2015;11(6):608–21.

3. Fan Z, Okello AA, Brooks DJ, et al. Longitudinal influence of microglial activation and amyloid on neuronal function in Alzheimer’s disease. Brain. 2015;138(12):3685–98.

4. Fan Z, Brooks DJ, Okello A, et al. An early and late peak in microglial activation in Alzheimer’s disease trajectory. Brain. 2017;140(3):792–803.

5. Yokokura M, Terada T, Bunai T, et al. Depiction of microglial activation in aging and dementia: positron emission tomography with [11C] DPA713 versus [11C](R) PK11195. Journal of Cerebral Blood Flow & Metabolism. 2017;37(3):877–89.

6. Parbo P, Ismail R, Hansen KV, et al. Brain inflammation accompanies amyloid in the majority of mild cognitive impairment cases due to Alzheimer’s disease. Brain. 2017;140(7):2002–11.

7. Parbo P, Ismail R, Sommerauer M, et al. Does inflammation precede tau aggregation in early Alzheimer’s disease? A PET study. Neurobiology of disease. 2018;117:211–6.

8. Passamonti L, Rodríguez PV, Hong YT, et al. [11C] PK11195 binding in Alzheimer disease and progressive supranuclear palsy. Neurology. 2018;90(22):e1989–96.

9. Parbo P, Madsen LS, Ismail R, et al. Low plasma neurofilament light levels associated with raised cortical microglial activation suggest inflammation acts to protect prodromal Alzheimer’s disease. Alzheimer’s research & therapy. 2020;12:1–7.

10. Ismail R, Parbo P, Madsen LS, et al. The relationships between neuroinflammation, beta-amyloid and tau deposition in Alzheimer’s disease: a longitudinal PET study. Journal of neuroinflammation. 2020;17:1–11.

11. Wang Q, Chen G, Schindler SE, et al. Baseline microglial activation correlates with brain amyloidosis and longitudinal cognitive decline in Alzheimer disease. Neurology: Neuroimmunology & Neuroinflammation. 2022;9(3):e1152.

12. Canário N, Jorge L, Martins R, et al. Dual PET-fMRI reveals a link between neuroinflammation, amyloid binding and compensatory task-related brain activity in Alzheimer’s disease. Communications biology. 2022;5(1):804.

13. Ekblad LL, Tuisku J, Koivumäki M, et al. Insulin resistance and body mass index are associated with TSPO PET in cognitively unimpaired elderly. Journal of Cerebral Blood Flow & Metabolism. 2023;43(9):1588–600.

14. Snellman A, Ekblad LL, Tuisku J, et al. APOE ε4 gene dose effect on imaging and blood biomarkers of neuroinflammation and beta-amyloid in cognitively unimpaired elderly. Alzheimer’s Research & Therapy. 2023;15(1):71.

15. Butler T, Wang X, Chiang G, et al. Reduction in Constitutively Activated Auditory Brainstem Microglia in Aging and Alzheimer’s Disease. Journal of Alzheimer’s Disease. 2024;99(1):307–19.

16. Madsen LS, Ismail R, Parbo P, et al. Microglial responses partially mediate the effect of Aβ on cognition in Alzheimer’s disease. Alzheimer’s & Dementia. 2024;20(11):8028–37.

17. Femminella GD, Ninan S, Atkinson R, et al. Does microglial activation influence hippocampal volume and neuronal function in Alzheimer’s disease and Parkinson’s disease dementia? Journal of Alzheimer’s Disease. 2016;51(4):1275–89.

18. Tondo G, Iaccarino L, Caminiti SP, et al. The combined effects of microglia activation and brain glucose hypometabolism in early-onset Alzheimer’s disease. Alzheimer’s Research & Therapy. 2020;12(1):50.

19. Ouchi Y, Yoshikawa E, Sekine Y, et al. Microglial activation and dopamine terminal loss in early Parkinson’s disease. Annals of Neurology. 2005 Feb;57(2):168–75.

20. Edison P, Ahmed I, Fan Z, et al. Microglia, Amyloid, and Glucose Metabolism in Parkinson’s Disease with and without Dementia. Neuropsychopharmacol. 2013 May;38(6):938–49.

21. Kaunzner UW, Kang Y, Zhang S, et al. Quantitative susceptibility mapping identifies inflammation in a subset of chronic multiple sclerosis lesions. Brain. 2019 Jan 1;142(1):133–45.

22. Nylund M, Sucksdorff M, Matilainen M, et al. Phenotyping of multiple sclerosis lesions according to innate immune cell activation using 18 kDa translocator protein-PET. Brain Communications. 2022 Jan 4;4(1):fcab301.

23. Rissanen E, Tuisku J, Rokka J, et al. In Vivo Detection of Diffuse Inflammation in Secondary Progressive Multiple Sclerosis Using PET Imaging and the Radioligand ^11^ C-PK11195. J Nucl Med. 2014 Jun;55(6):939–44.

24. Laaksonen S, Saraste M, Sucksdorff M, et al. Early prognosticators of later TSPO-PET-measurable microglial activation in multiple sclerosis. Multiple Sclerosis and Related Disorders. 2023 Jul;75:104755.

25. Misin O, Matilainen M, Nylund M, et al. Innate Immune Cell–Related Pathology in the Thalamus Signals a Risk for Disability Progression in Multiple Sclerosis. Neurol Neuroimmunol Neuroinflamm. 2022 Jul;9(4):e1182.

26. Sucksdorff M, Matilainen M, Tuisku J, et al. Brain TSPO-PET predicts later disease progression independent of relapses in multiple sclerosis. Brain. 2020 Nov 1;143(11):3318–30.

27. Sucksdorff M, Rissanen E, Tuisku J, et al. Evaluation of the Effect of Fingolimod Treatment on Microglial Activation Using Serial PET Imaging in Multiple Sclerosis. J Nucl Med. 2017 Oct;58(10):1646–51.

28. Bezukladova S, Tuisku J, Matilainen M, et al. Insights into disseminated MS brain pathology with multimodal diffusion tensor and PET imaging. Neurol Neuroimmunol Neuroinflamm. 2020 May;7(3):e691.

29. Sucksdorff M, Tuisku J, Matilainen M, et al. Natalizumab treatment reduces microglial activation in the white matter of the MS brain. Neurol Neuroimmunol Neuroinflamm. 2019 Jul;6(4):e574.

30. Lehto J, Nylund M, Matilainen M, et al. Longitudinal stability of progression‐related microglial activity during teriflunomide treatment in patients with multiple sclerosis. Euro J of Neurology. 2023 Aug;30(8):2365–75.

31. Pitombeira MS, Koole M, Campanholo KR, et al. Innate immune cells and myelin profile in multiple sclerosis: a multi-tracer PET/MR study. Eur J Nucl Med Mol Imaging. 2022 Nov;49(13):4551–66.

32. Laaksonen S, Saraste M, Nylund M, et al. Sex-driven variability in TSPO-expressing microglia in MS patients and healthy individuals. Front Neurol. 2024 Feb 20;15:1352116.

33. Saraste M, Matilainen M, Rajda C, et al. Association between microglial activation and serum kynurenine pathway metabolites in multiple sclerosis patients. Multiple Sclerosis and Related Disorders. 2022 Mar;59:103667.

34. Kang Y, Pandya S, Zinger N, et al. Longitudinal change in TSPO PET imaging in progressive multiple sclerosis. Ann Clin Transl Neurol. 2021 Aug;8(8):1755–9.

35. Politis M, Lahiri N, Niccolini F, et al. Increased central microglial activation associated with peripheral cytokine levels in premanifest Huntington’s disease gene carriers. Neurobiology of disease. 2015;83:115–21.

36. Doorduin J, De Vries EFJ, Willemsen ATM, et al. Neuroinflammation in Schizophrenia-Related Psychosis: A PET Study. J Nucl Med. 2009 Nov;50(11):1801–7.

37. Jonker I, Doorduin J, Knegtering H, et al. Antiviral treatment in schizophrenia: a randomized pilot PET study on the effects of valaciclovir on neuroinflammation. Psychol Med. 2023 Nov;53(15):7087–95.

38. Conen S, Gregory CJ, Hinz R, et al. Neuroinflammation as measured by positron emission tomography in patients with recent onset and established schizophrenia: implications for immune pathogenesis. Mol Psychiatry. 2021 Sep;26(9):5398–406.

39. Di Biase MA, Zalesky A, O’keefe G, et al. PET imaging of putative microglial activation in individuals at ultra-high risk for psychosis, recently diagnosed and chronically ill with schizophrenia. Transl Psychiatry. 2017 Aug 29;7(8):e1225–e1225.

40. Schubert JJ, Veronese M, Fryer TD, et al. A modest increase in 11C-PK11195-positron emission tomography TSPO binding in depression is not associated with serum C-reactive protein or body mass index. Biological Psychiatry: Cognitive Neuroscience and Neuroimaging. 2021;6(7):716–24.

41. Su L, Faluyi YO, Hong YT, et al. Neuroinflammatory and morphological changes in late-life depression: the NIMROD study. The British journal of psychiatry. 2016;209(6):525–6.

42. Holmes SE, Hinz R, Conen S, et al. Elevated translocator protein in anterior cingulate in major depression and a role for inflammation in suicidal thinking: a positron emission tomography study. Biological psychiatry. 2018;83(1):61–9.

43. Joo YH, Lee MW, Son YD, et al. In vivo cerebral translocator protein (Tspo) binding and its relationship with blood adiponectin levels in treatment-naïve young adults with major depression: a [11c] pk11195 pet study. Biomedicines. 2021;10(1):34.

44. Lyoo CH, Ikawa M, Liow JS, et al. Cerebellum can serve as a pseudo-reference region in Alzheimer disease to detect neuroinflammation measured with PET radioligand binding to translocator protein. Journal of Nuclear Medicine. 2015;56(5):701–6.

45. Kreisl WC, Lyoo CH, McGwier M, et al. In vivo radioligand binding to translocator protein correlates with severity of Alzheimer’s disease. Brain. 2013;136(7):2228–38.

46. Kreisl WC, Lyoo CH, Liow JS, et al. 11C-PBR28 binding to translocator protein increases with progression of Alzheimer’s disease. Neurobiology of aging. 2016;44:53–61.

47. Toppala S, Ekblad LL, Tuisku J, et al. Association of early β-amyloid accumulation and neuroinflammation measured with [11C] PBR28 in elderly individuals without dementia. Neurology. 2021;96(12):e1608–19.

48. Dani M, Wood M, Mizoguchi R, et al. Microglial activation correlates in vivo with both tau and amyloid in Alzheimer’s disease. Brain. 2018;141(9):2740–54.

49. Rossano SM, Johnson AS, Smith A, et al. Microglia measured by TSPO PET are associated with Alzheimer’s disease pathology and mediate key steps in a disease progression model. Alzheimer’s & Dementia. 2024;20(4):2397–407.

50. Zou J, Tao S, Johnson A, et al. Microglial activation, but not tau pathology, is independently associated with amyloid positivity and memory impairment. Neurobiology of aging. 2020;85:11–21.

51. Pascoal TA, Benedet AL, Ashton NJ, et al. Microglial activation and tau propagate jointly across Braak stages. Nature medicine. 2021;27(9):1592–9.

52. Su L, Surendranathan A, Huang Y, et al. Relationship between tau, neuroinflammation and atrophy in Alzheimer’s disease: The NIMROD study. Information Fusion. 2021;67:116–24.

53. Kreisl WC, Lyoo CH, Liow JS, et al. Distinct patterns of increased translocator protein in posterior cortical atrophy and amnestic Alzheimer’s disease. Neurobiology of aging. 2017;51:132–40.

54. Femminella GD, Dani M, Wood M, et al. Microglial activation in early Alzheimer trajectory is associated with higher gray matter volume. Neurology. 2019;92(12):e1331–43.

55. Varnäs K, Cselényi Z, Jucaite A, et al. PET imaging of [11C]PBR28 in Parkinson’s disease patients does not indicate increased binding to TSPO despite reduced dopamine transporter binding. Eur J Nucl Med Mol Imaging. 2019 Feb;46(2):367–75.

56. Laurell GL, Plavén-Sigray P, Jucaite A, et al. Nondisplaceable Binding Is a Potential Confounding Factor in ^11^ C-PBR28 Translocator Protein PET Studies. J Nucl Med. 2021 Mar;62(3):412–7.

57. Jucaite A, Svenningsson P, Rinne JO, et al. Effect of the myeloperoxidase inhibitor AZD3241 on microglia: a PET study in Parkinson’s disease. Brain. 2015 Sep;138(9):2687–700.

58. Al-Abdulrasul H, Ajalin R, Tuisku J, et al. Neuroinflammation in Parkinson’s disease: A study with [11C]PBR28 PET and cerebrospinal fluid markers. Parkinsonism & Related Disorders. 2025 Jan;130:107177.

59. Gershen LD. Neuroinflammation in Temporal Lobe Epilepsy Measured Using Positron Emission Tomographic Imaging of Translocator Protein (vol 72, pg 882, 2015). JAMA NEUROLOGY. 2015;72(8):950–950.

60. Dickstein LP, Liow J, Austermuehle A, et al. Neuroinflammation in neocortical epilepsy measured by PET imaging of translocator protein. Epilepsia. 2019 Jun;60(6):1248–54.

61. Park E, Gallezot JD, Delgadillo A, et al. 11C-PBR28 imaging in multiple sclerosis patients and healthy controls: test-retest reproducibility and focal visualization of active white matter areas. Eur J Nucl Med Mol Imaging. 2015 Jun;42(7):1081–92.

62. Datta G, Colasanti A, Rabiner EA, et al. Neuroinflammation and its relationship to changes in brain volume and white matter lesions in multiple sclerosis. Brain. 2017 Nov 1;140(11):2927–38.

63. Herranz E, Giannì C, Louapre C, et al. Neuroinflammatory component of gray matter pathology in multiple sclerosis. Annals of Neurology. 2016 Nov;80(5):776–90.

64. Datta G, Colasanti A, Kalk N, et al. ^11^ C-PBR28 and ^18^ F-PBR111 Detect White Matter Inflammatory Heterogeneity in Multiple Sclerosis. J Nucl Med. 2017 Sep;58(9):1477–82.

65. Herranz E, Treaba CA, Barletta VT, et al. Characterization of cortico-meningeal translocator protein expression in multiple sclerosis. Brain. 2024 Jul 5;147(7):2566–78.

66. Datta G, Violante IR, Scott G, et al. Translocator positron-emission tomography and magnetic resonance spectroscopic imaging of brain glial cell activation in multiple sclerosis. Multiple Sclerosis Journal. 2017;23(11):1469–78.

67. Lois C, González I, Izquierdo-García D, et al. Neuroinflammation in Huntington’s Disease: New Insights with ^11^ C-PBR28 PET/MRI. ACS Chem Neurosci. 2018 Nov 21;9(11):2563–71.

68. Roussakis AA, Gennaro M, Gordon MF, et al. A PET-CT study on neuroinflammation in Huntington’s disease patients participating in a randomized trial with laquinimod. Brain Communications. 2023;5(2):fcad084.

69. Selvaraj S, Bloomfield PS, Cao B, et al. Brain TSPO imaging and gray matter volume in schizophrenia patients and in people at ultra high risk of psychosis: An [11C]PBR28 study. Schizophrenia Research. 2018 May;195:206–14.

70. Bloomfield PS, Selvaraj S, Veronese M, et al. Microglial Activity in People at Ultra High Risk of Psychosis and in Schizophrenia: An [ ^11^ C]PBR28 PET Brain Imaging Study. AJP. 2016 Jan;173(1):44–52.

71. Holmes SE, Hinz R, Drake RJ, et al. In vivo imaging of brain microglial activity in antipsychotic-free and medicated schizophrenia: a [11C](R)-PK11195 positron emission tomography study. Mol Psychiatry. 2016 Dec;21(12):1672–9.

72. Richards EM, Zanotti-Fregonara P, Fujita M, et al. PET radioligand binding to translocator protein (TSPO) is increased in unmedicated depressed subjects. EJNMMI Res. 2018 Dec;8(1):57.

73. Hannestad J, DellaGioia N, Gallezot JD, et al. The neuroinflammation marker translocator protein is not elevated in individuals with mild-to-moderate depression: a [11C] PBR28 PET study. Brain, behavior, and immunity. 2013;33:131–8.

74. Varrone A, Oikonen V, Forsberg A, et al. Positron emission tomography imaging of the 18-kDa translocator protein (TSPO) with [18 F] FEMPA in Alzheimer’s disease patients and control subjects. European Journal of Nuclear Medicine and Molecular Imaging. 2015;42:438–46.

75. Lee H, Noh Y, Kim WR, et al. Translocator protein (18 kDa) polymorphism (rs6971) in the Korean population. Dementia and Neurocognitive Disorders. 2022;21(2):71.

76. Zhang M, Qian X hang, Hu J, et al. Integrating TSPO PET imaging and transcriptomics to unveil the role of neuroinflammation and amyloid-β deposition in Alzheimer’s disease. European journal of nuclear medicine and molecular imaging. 2024;51(2):455–67.

77. Hamelin L, Lagarde J, Dorothée G, et al. Early and protective microglial activation in Alzheimer’s disease: a prospective study using 18 F-DPA-714 PET imaging. Brain. 2016;139(4):1252–64.

78. Hamelin L, Lagarde J, Dorothée G, et al. Distinct dynamic profiles of microglial activation are associated with progression of Alzheimer’s disease. Brain. 2018;141(6):1855–70.

79. Golla SS, Boellaard R, Oikonen V, et al. Quantification of [18F] DPA-714 binding in the human brain: initial studies in healthy controls and Alzheimer’s disease patients. Journal of Cerebral Blood Flow & Metabolism. 2015;35(5):766–72.

80. Gouilly D, Salabert AS, Bertrand E, et al. Clinical heterogeneity of neuro-inflammatory PET profiles in early Alzheimer’s disease. Frontiers in Neurology. 2023;14:1189278.

81. Cousins O, Schubert JJ, Chandra A, et al. Microglial activation, tau and amyloid deposition in TREM2 p.R47H carriers and mild cognitive impairment patients: a multi-modal/multi-tracer PET/MRI imaging study with influenza vaccine immune challenge. J Neuroinflammation. 2023 Nov 21;20(1):272.

82. Lavisse S, Goutal S, Wimberley C, et al. Increased microglial activation in patients with Parkinson disease using [18F]-DPA714 TSPO PET imaging. Parkinsonism & Related Disorders. 2021 Jan;82:29–36.

83. Yacoubian TA, Fang YD, Gerstenecker A, et al. Brain and Systemic Inflammation in De Novo Parkinson’s Disease. Movement Disorders. 2023 May;38(5):743–54.

84. Hagens MHJ, Golla SV, Wijburg MT, et al. In vivo assessment of neuroinflammation in progressive multiple sclerosis: a proof of concept study with [18F]DPA714 PET. J Neuroinflammation. 2018 Dec;15(1):314.

85. Bodini B, Poirion E, Tonietto M, et al. Individual Mapping of Innate Immune Cell Activation Is a Candidate Marker of Patient-Specific Trajectories of Worsening Disability in Multiple Sclerosis. J Nucl Med. 2020 Jul;61(7):1043–9.

86. Hamzaoui M, Garcia J, Boffa G, et al. Positron Emission Tomography with [ ^18^ F ]‐ DPA ‐714 Unveils a Smoldering Component in Most Multiple Sclerosis Lesions which Drives Disease Progression. Annals of Neurology. 2023 Aug;94(2):366–83.

87. Poirion E, Tonietto M, Lejeune FX, et al. Structural and Clinical Correlates of a Periventricular Gradient of Neuroinflammation in Multiple Sclerosis. Neurology [Internet]. 2021 Apr 6 [cited 2024 Apr 25];96(14). Available from: https://www.neurology.org/doi/10.1212/WNL.0000000000011700

88. Ricigliano VAG, Morena E, Colombi A, et al. Choroid Plexus Enlargement in Inflammatory Multiple Sclerosis: 3.0-T MRI and Translocator Protein PET Evaluation. Radiology. 2021 Oct;301(1):166–77.

89. Ricigliano VAG, Louapre C, Poirion E, et al. Imaging Characteristics of Choroid Plexuses in Presymptomatic Multiple Sclerosis: A Retrospective Study. Neurol Neuroimmunol Neuroinflamm. 2022 Nov;9(6):e200026.

90. Bunai T, Terada T, Kono S, et al. Neuroinflammation following disease modifying therapy in multiple sclerosis: A pilot positron emission tomography study. Journal of the Neurological Sciences. 2018 Feb;385:30–3.

91. Yasuno F, Watanabe A, Kimura Y, et al. Estimation of blood-based biomarkers of glial activation related to neuroinflammation. Brain, Behavior, & Immunity-Health. 2022;26:100549.

92. Yasuno F, Kimura Y, Ogata A, et al. Trait-anxiety and glial-related neuroinflammation of the amygdala and its associated regions in Alzheimer’s disease: A significant correlation. Brain, Behavior, & Immunity-Health. 2024;38:100795.

93. Terada T, Yokokura M, Obi T, et al. In vivo direct relation of tau pathology with neuroinflammation in early Alzheimer’s disease. Journal of neurology. 2019;266:2186–96.

94. Yasuno F, Kimura Y, Ogata A, et al. Neuroimaging biomarkers of glial activation for predicting the annual cognitive function decline in patients with Alzheimer’s disease. Brain, Behavior, and Immunity. 2023;114:214–20.

95. Akerele MI, Zein SA, Pandya S, et al. Population-based input function for TSPO quantification and kinetic modeling with [11C]-DPA-713. EJNMMI Phys. 2021 Dec;8(1):39.

96. Kagitani-Shimono K, Kato H, Kuwayama R, et al. Clinical evaluation of neuroinflammation in child-onset focal epilepsy: a translocator protein PET study. J Neuroinflammation. 2021 Dec;18(1):8.

97. Kagitani-Shimono K, Kato H, Soeda F, et al. Extension of microglial activation is associated with epilepsy and cognitive dysfunction in Tuberous sclerosis complex: A TSPO-PET study. NeuroImage: Clinical. 2023;37:103288.

98. Coughlin JM, Wang Y, Ambinder EB, et al. In vivo markers of inflammatory response in recent-onset schizophrenia: a combined study using [11C]DPA-713 PET and analysis of CSF and plasma. Transl Psychiatry. 2016 Apr 12;6(4):e777–e777.

99. Knezevic D, Verhoeff NPL, Hafizi S, et al. Imaging microglial activation and amyloid burden in amnestic mild cognitive impairment. Journal of Cerebral Blood Flow & Metabolism. 2018;38(11):1885–95.

100. Suridjan I, Pollock B, Verhoeff N, et al. In-vivo imaging of grey and white matter neuroinflammation in Alzheimer’s disease: a positron emission tomography study with a novel radioligand,[18F]-FEPPA. Molecular psychiatry. 2015;20(12):1579–87.

101. Cisbani G. Peripheral cytokine and fatty acid associations with neuroinflammation in AD and aMCI patients: an exploratory study. In WILEY 111 RIVER ST, HOBOKEN 07030-5774, NJ USA; 2021. p. 263–4.

102. Ghadery C, Koshimori Y, Coakeley S, et al. Microglial activation in Parkinson’s disease using [18F]-FEPPA. J Neuroinflammation. 2017 Dec;14(1):8.

103. Mabrouk R, Strafella AP, Knezevic D, et al. Feasibility study of TSPO quantification with [18F]FEPPA using population-based input function. Garg P, editor. PLoS ONE. 2017 May 17;12(5):e0177785.

104. Koshimori Y, Ko JH, Mizrahi R, et al. Imaging Striatal Microglial Activation in Patients with Parkinson’s Disease. Tansey MG, editor. PLoS ONE. 2015 Sep 18;10(9):e0138721.

105. Ghadery C, Koshimori Y, Christopher L, et al. The Interaction Between Neuroinflammation and β-Amyloid in Cognitive Decline in Parkinson’s Disease. Mol Neurobiol. 2020 Jan;57(1):492–501.

106. Kilmer J, Rodrigo S, Petrescu A, et al. TSPO ‐ PET in pre‐surgical evaluations: Correlation of neuroinflammation and SEEG epileptogenicity mapping in drug‐resistant focal epilepsy. Epilepsia. 2025 Feb;66(2):430–43.

107. Cheval M, Rodrigo S, Taussig D, et al. [^18^ F]DPA-714 PET Imaging in the Presurgical Evaluation of Patients With Drug-Resistant Focal Epilepsy. Neurology [Internet]. 2023 Nov 7 [cited 2025 Mar 26];101(19). Available from: https://www.neurology.org/doi/10.1212/WNL.0000000000207811

108. Wang J, Ge J, Jin L, et al. Characterization of neuroinflammation pattern in anti-LGI1 encephalitis based on TSPO PET and symptom clustering analysis. Eur J Nucl Med Mol Imaging. 2023 Jul;50(8):2394–408.

109. Kenk M, Selvanathan T, Rao N, et al. Imaging Neuroinflammation in Gray and White Matter in Schizophrenia: An In-Vivo PET Study With [18F]-FEPPA. Schizophrenia Bulletin. 2015 Jan 1;41(1):85–93.

110. Setiawan E, Wilson AA, Mizrahi R, et al. Role of translocator protein density, a marker of neuroinflammation, in the brain during major depressive episodes. JAMA psychiatry. 2015;72(3):268–75.

111. Braga J, Lepra M, Kish SJ, et al. Neuroinflammation after COVID-19 with persistent depressive and cognitive symptoms. JAMA psychiatry. 2023;80(8):787–95.

112. Li H, Sagar AP, Kéri S. Microglial markers in the frontal cortex are related to cognitive dysfunctions in major depressive disorder. Journal of Affective Disorders. 2018;241:305–10.

113. Setiawan E, Attwells S, Wilson AA, et al. Association of translocator protein total distribution volume with duration of untreated major depressive disorder: a cross-sectional study. The Lancet Psychiatry. 2018;5(4):339–47.

114. Attwells S, Setiawan E, Rusjan PM, et al. Translocator protein distribution volume predicts reduction of symptoms during open-label trial of celecoxib in major depressive disorder. Biological Psychiatry. 2020;88(8):649–56.

115. Attwells S, Setiawan E, Rusjan PM, et al. A double-blind placebo-controlled trial of minocycline on translocator protein distribution volume in treatment-resistant major depressive disorder. Translational Psychiatry. 2021;11(1):334.

116. Li H, Sagar AP, Kéri S. Translocator protein (18 kDa TSPO) binding, a marker of microglia, is reduced in major depression during cognitive-behavioral therapy. Progress in Neuro-Psychopharmacology and Biological Psychiatry. 2018;83:1–7.

117. Attwells S, Setiawan E, Wilson AA, et al. Replicating predictive serum correlates of greater translocator protein distribution volume in brain. Neuropsychopharmacology. 2020;45(6):925–31.

118. Varrone A, Mattsson P, Forsberg A, et al. In vivo imaging of the 18-kDa translocator protein (TSPO) with [18 F] FEDAA1106 and PET does not show increased binding in Alzheimer’s disease patients. European journal of nuclear medicine and molecular imaging. 2013;40:921–31.

119. Takano A, Piehl F, Hillert J, et al. In vivo TSPO imaging in patients with multiple sclerosis: a brain PET study with [18F] FEDAA1106. EJNMMI research. 2013;3(1):30.

120. Colasanti A, Guo Q, Muhlert N, et al. In Vivo Assessment of Brain White Matter Inflammation in Multiple Sclerosis with ^18^ F-PBR111 PET. J Nucl Med. 2014 Jul;55(7):1112–8.

121. Colasanti A, Guo Q, Giannetti P, et al. Hippocampal neuroinflammation, functional connectivity, and depressive symptoms in multiple sclerosis. Biological psychiatry. 2016;80(1):62–72.

122. Singhal T, Cicero S, Rissanen E, et al. Glial Activity Load on PET Reveals Persistent “Smoldering” Inflammation in MS Despite Disease-Modifying Treatment: 18F-PBR06 Study. Clin Nucl Med. 2024 Jun;49(6):491–9.

123. Singhal T, O’Connor K, Dubey S, et al. 18F-PBR06 Versus 11C-PBR28 PET for Assessing White Matter Translocator Protein Binding in Multiple Sclerosis. Clin Nucl Med. 2018 Sep;43(9):e289–95.

124. Singhal T, O’Connor K, Dubey S, et al. Gray matter microglial activation in relapsing vs progressive MS: A [F-18]PBR06-PET study. Neurol Neuroimmunol Neuroinflamm. 2019 Sep;6(5):e587.

125. Biechele G, Rauchmann BS, Janowitz D, et al. Associations between sex, body mass index and the individual microglial response in Alzheimer’s disease. Journal of neuroinflammation. 2024;21(1):30.

126. Rauchmann B, Brendel M, Franzmeier N, et al. Microglial activation and connectivity in Alzheimer disease and aging. Annals of neurology. 2022;92(5):768–81.

127. Finze A, Biechele G, Rauchmann BS, et al. Individual regional associations between Aβ-, tau-and neurodegeneration (ATN) with microglial activation in patients with primary and secondary tauopathies. Molecular psychiatry. 2023;28(10):4438–50.

128. Unterrainer M, Mahler C, Vomacka L, et al. TSPO PET with [18F]GE-180 sensitively detects focal neuroinflammation in patients with relapsing–remitting multiple sclerosis. Eur J Nucl Med Mol Imaging. 2018 Jul;45(8):1423–31.

129. Appleton J, Finn Q, Zanotti-Fregonara P, et al. Brain inflammation co-localizes highly with tau in mild cognitive impairment due to early-onset Alzheimer’s disease. Brain. 2025;148(1):119–32.

130. Rocha NP, Charron O, Latham LB, et al. Microglia Activation in Basal Ganglia Is a Late Event in Huntington Disease Pathophysiology. Neurol Neuroimmunol Neuroinflamm. 2021 May;8(3):e984.

131. Herzog S, Bartlett EA, Zanderigo F, et al. Neuroinflammation, Stress-Related Suicidal Ideation, and Negative Mood in Depression. JAMA psychiatry. 2025;82(1):85–93.

132. Montagne A, Barnes SR, Sweeney MD, et al. Blood-brain barrier breakdown in the aging human hippocampus. Neuron. 2015;85(2):296–302.

133. Denkinger M, Baker S, Inglis B, et al. Associations between regional blood-brain barrier permeability, aging, and Alzheimer’s disease biomarkers in cognitively normal older adults. Plos one. 2024;19(6):e0299764.

134. Nation DA, Sweeney MD, Montagne A, et al. Blood–brain barrier breakdown is an early biomarker of human cognitive dysfunction. Nature medicine. 2019;25(2):270–6.

135. Preis L, Villringer K, Brosseron F, et al. Assessing blood-brain barrier dysfunction and its association with Alzheimer’s pathology, cognitive impairment and neuroinflammation. Alz Res Therapy. 2024 Jul 31;16(1):172.

136. Montagne A, Nation DA, Sagare AP, et al. APOE4 leads to blood–brain barrier dysfunction predicting cognitive decline. Nature. 2020;581(7806):71–6.

137. Reas ET, Solders SK, Tsiknia A, et al. *APOE* 𝜀4‐related blood–brain barrier breakdown is associated with microstructural abnormalities. Alzheimer’s &amp; Dementia. 2024 Dec;20(12):8615–24.

138. Li M, Li Y, Zuo L, et al. Increase of blood-brain barrier leakage is related to cognitive decline in vascular mild cognitive impairment. Bmc Neurology. 2021;21(1):1–8.

139. Moon Y, Jeon HJ, Han SH, et al. Blood-brain barrier breakdown is linked to tau pathology and neuronal injury in a differential manner according to amyloid deposition. J Cereb Blood Flow Metab. 2023 Nov;43(11):1813–25.

140. Moon Y, Lim C, Kim Y, et al. Sex-related differences in regional blood–brain barrier integrity in non-demented elderly subjects. International Journal of Molecular Sciences. 2021;22(6):2860.

141. Choi JD, Moon Y, Kim HJ, et al. Choroid plexus volume and permeability at brain MRI within the Alzheimer disease clinical spectrum. Radiology. 2022;304(3):635–45.

142. Alruwais NM, Rusted JM, Tabet N, et al. Evidence of emerging BBB changes in mid‐age apolipoprotein E epsilon‐4 carriers. Brain and Behavior. 2022;12(12):e2806.

143. Al-Bachari S, Naish JH, Parker GJM, et al. Blood–Brain Barrier Leakage Is Increased in Parkinson’s Disease. Front Physiol. 2020 Dec 22;11:593026.

144. Gupta RK, Awasthi R, Garg RK, et al. T1-weighted dynamic contrast-enhanced MR evaluation of different stages of neurocysticercosis and its relationship with serum MMP-9 expression. American Journal of Neuroradiology. 2013;34(5):997–1003.

145. Li H, Liu X, Wang R, et al. Blood–brain barrier damage and new onset refractory status epilepticus: An exploratory study using dynamic contrast‐enhanced magnetic resonance imaging. Epilepsia. 2023 Jun;64(6):1594–604.

146. Singh AK, Garg RK, Gupta RK, et al. Dynamic contrast-enhanced (DCE) MRI derived kinetic perfusion indices may help predicting seizure control in single calcified neurocysticercosis. Magnetic Resonance Imaging. 2018;49:55–62.

147. Oghabian MA, Fatemidokht A, Haririchian MH. Quantification of blood-brain-barrier permeability dysregulation and inflammatory activity in ms lesions by dynamic-contrast enhanced MR imaging. Basic and Clinical Neuroscience. 2022;13(1):117.

148. Xiong H, Yin P, Li X, et al. The features of cerebral permeability and perfusion detected by dynamic contrast-enhanced magnetic resonance imaging with Patlak model in relapsing&ndash;remitting multiple sclerosis. TCRM. 2019 Feb;Volume 15:233–40.

149. Cramer SP, Simonsen H, Frederiksen JL, et al. Abnormal blood–brain barrier permeability in normal appearing white matter in multiple sclerosis investigated by MRI. NeuroImage: Clinical. 2014;4:182–9.

150. Cheng Y, Wang T, Zhang T, et al. Increased Blood-Brain Barrier Permeability of the Thalamus Correlated With Symptom Severity and Brain Volume Alterations in Patients With Schizophrenia. Biological Psychiatry: Cognitive Neuroscience and Neuroimaging. 2022 Oct;7(10):1025–34.

151. van de Haar HJ, Jansen JFA, van Osch MJP, et al. Neurovascular unit impairment in early Alzheimer’s disease measured with magnetic resonance imaging. Neurobiology of Aging. 2016 Sep 1;45:190–6.

152. Freeze WM, Jacobs HI, De Jong JJ, et al. White matter hyperintensities mediate the association between blood-brain barrier leakage and information processing speed. Neurobiology of Aging. 2020;85:113–22.

153. Van De Haar HJ, Burgmans S, Jansen JF, et al. Blood-brain barrier leakage in patients with early Alzheimer disease. Radiology. 2016;281(2):527–35.

154. Verheggen IC, de Jong JJ, van Boxtel MP, et al. Imaging the role of blood–brain barrier disruption in normal cognitive ageing. Geroscience. 2020;42:1751–64.

155. Varatharaj A, Liljeroth M, Darekar A, et al. Blood–brain barrier permeability measured using dynamic contrast‐enhanced magnetic resonance imaging: a validation study. The Journal of Physiology. 2019 Feb;597(3):699–709.

156. Cramer SP, Simonsen HJ, Varatharaj A, et al. Permeability of the blood–brain barrier predicts no evidence of disease activity at 2 years after natalizumab or fingolimod treatment in relapsing–remitting multiple sclerosis. Annals of Neurology. 2018 May;83(5):902–14.

157. Knudsen MH, Lindberg U, Frederiksen JL, et al. Blood-brain barrier permeability changes in the first year after alemtuzumab treatment predict 2-year outcomes in relapsing-remitting multiple sclerosis. Multiple Sclerosis and Related Disorders. 2022 Jul;63:103891.

158. Ingrisch M, Sourbron S, Herberich S, et al. Dynamic Contrast-Enhanced Magnetic Resonance Imaging Suggests Normal Perfusion in Normal-Appearing White Matter in Multiple Sclerosis. Invest Radiol. 2017 Mar;52(3):135–41.

159. Hergert DC, Gaasedelen O, Ryman SG, et al. Blood–Brain Barrier Permeability Is Associated With Cognitive Functioning in Normal Aging and Neurodegenerative Diseases. JAHA. 2024 Jul 16;13(14):e034225.

160. Cafri N, Mirloo S, Zarhin D, et al. Imaging blood–brain barrier dysfunction in drug‐resistant epilepsy: A multi‐center feasibility study. Epilepsia. 2025;66(1):195–206.
